# *Vitis vinifera* seed extract reduces venous reflux time in patients with varicose veins: VICTORY randomized controlled trial

**DOI:** 10.1016/j.jvsv.2025.102355

**Published:** 2025-11-15

**Authors:** SungA Bae, Hakju Kim, Nak-Hoon Son, Minkwan Kim, Sungjoon Park, In Hyun Jung

**Affiliations:** a Department of Cardiology, Yongin Severance Hospital, Yonsei University College of Medicine, Yongin, Republic of Korea; b Department of Thoracic and Cardiovascular Surgery, Yongin Severance Hospital, Yonsei University College of Medicine, Yongin, Republic of Korea; c Department of Statistics, Keimyung University, Daegu, Republic of Korea

**Keywords:** Deep vein insufficiency, Quality of life, Randomized controlled trial, Varicose veins, Venous reflux, *Vitis vinifera* seed extract

## Abstract

**Background:**

Varicose veins affect 25% to 40% of adults, with higher prevalence in older populations. Although *Vitis vinifera* seed extract (VVSE) can alleviate symptoms of venous insufficiency, its effects on venous reflux remain uncertain. We hypothesized that oral VVSE supplementation would significantly reduce venous reflux time compared with lifestyle modifications alone in patients with lower extremity varicose veins.

**Methods:**

This prospective, open-label, randomized controlled trial enrolled adults aged 19 to 80 years with varicose veins and venous reflux confirmed by Doppler ultrasound (≥0.5 seconds in superficial veins or ≥1 second in deep veins). Patients were provided oral VVSE (150 mg twice daily) plus therapeutic lifestyle changes or therapeutic lifestyle changes alone (control) over 12 weeks. The mean venous reflux time, changes in specific vein reflux times, proportion achieving improved reflux (≤0.5 seconds for superficial veins, ≤1 second for deep veins), Venous Clinical Severity Score (VCSS), and Chronic Venous Insufficiency Quality of Life Questionnaire (CIVIQ-14) score were compared.

**Results:**

Among 176 participants (88/group), the VVSE group experienced significantly greater reductions in mean venous reflux time. Improvements were observed in both superficial and deep veins, with notable effects in the great saphenous and popliteal veins. The proportion of patients achieving improved venous reflux was significantly higher in the VVSE group. The VVSE group also demonstrated superior symptom improvement, with greater reductions in VCSS and CIVIQ-14 scores.

**Conclusions:**

VVSE significantly reduced venous reflux time across both superficial and deep veins, improving patient-reported symptoms compared with lifestyle modifications alone. The beneficial effects were consistent across diverse patient subgroups and particularly noteworthy in deep venous insufficiency.

**Article Highlights**

• **Type of Research:** Randomized controlled trial
• **Key Findings:** Patients receiving *Vitis vinifera* seed extract (VVSE) for 12 weeks showed significantly greater reductions in venous reflux time compared with controls. Symptom scores (Venous Clinical Severity Score and CIVIQ-14) also improved significantly in the VVSE group, demonstrating parallel enhancements in both objective hemodynamic parameters and patient-reported symptoms.
• **Take Home Message:** This study provides the first objective evidence using Doppler ultrasonography that VVSE can reduce venous reflux time in patients with varicose veins. Multicenter, placebo-controlled trials are warranted to confirm these findings.

Varicose veins are characterized by venous valve incompetence in the lower extremity circulation, which leads to pathological retrograde blood flow (venous reflux), causing progressive venous dilation, elongation, and tortuosity of the affected vessels.^1^ This condition impacts superficial veins, including the great saphenous, small saphenous, and perforating veins.^2^ This retrograde flow causes patients to experience leg pain and other symptoms, but patients most often seek medical attention because of noticeable vein enlargement.^3^

Between 25% and 40% of adults worldwide suffer from varicose veins, whereas individuals over 60 years old experience an increased occurrence rate of 57% to 63.2%.^4^^,^^5^ Western and developed countries see higher incidences of this condition, whereas aging populations and lifestyle shifts will likely lead to increased prevalence rates.^6^ The primary risk factors for this condition are older age, female sex (due to hormonal changes associated with multiple pregnancies), obesity, and extended periods of standing.^7^ Untreated varicose veins may lead to complications including skin pigmentation, edema, and venous ulcers, underscoring the importance of early intervention in populations at high risk.^8^^,^^9^

The primary diagnostic approach uses Doppler ultrasonography to detect reflux in both superficial and deep veins.^10^ Treatment options range from noninvasive lifestyle changes and compression stockings to invasive procedures like radiofrequency or laser ablation, which focus on eradicating venous reflux.^11^^,^^12^

*Vitis vinifera* seed extract (VVSE) contains proanthocyanidins, which are oligomeric flavonoid compounds with potent antioxidant and vascular protective properties. Proanthocyanidins exhibit capillary-stabilizing effects, reduce vascular permeability, and may strengthen venous walls by preserving endothelial integrity and inhibiting collagen and elastin-degrading enzymes.^13^ These water-soluble compounds are naturally found in various fruits and have demonstrated a favorable safety profile with minimal side effects.^14^ Due to these vascular-specific mechanisms, VVSE containing proanthocyanidins represents a hopeful noninvasive treatment option for patients with venous and lymphatic insufficiency.^15^ VVSE provides relief for leg pain as well as heaviness and swelling, which often occur during extended periods of sitting.^16^^,^^17^ Among other venoactive drugs, red vine leaf extract (AS 195) has shown efficacy in improving signs and symptoms of chronic venous insufficiency,^18^ whereas micronized purified flavonoid fraction has demonstrated clinical benefits including reduction in venous reflux time in randomized controlled trials.^19^ Recent comparative studies have shown VVSE to be noninferior to micronized purified flavonoid fraction for symptomatic relief in chronic venous disease.^20^ However, although symptomatic benefits have been established, direct evidence demonstrating VVSE’s effects on objective hemodynamic parameters such as venous reflux time remains limited.

We hypothesized that oral VVSE supplementation would significantly reduce venous reflux time and improve clinical symptoms compared with lifestyle modifications alone in patients with varicose veins. Therefore, the present study evaluated this hypothesis using objective Doppler ultrasonography measurements and validated clinical assessment tools.

## Methods

### Study design and participants

This study was an investigator-initiated trial conducted at a single center, and designed as an open-label randomized control study using *Vitis vinifera* seed extract for treating varicose veins in lower extremities (VICTORY trial; ClinicalTrials.gov identifier: NCT05851183). The trial protocol was approved by the institutional review board of Yonsei University Yongin Severance Hospital under approval number 9-2023-0031. All protocols followed the Declaration of Helsinki guidelines. Before enrolment, each patient signed a written informed consent document. The appendix contains the final study protocol in the Supplementary Materials (online only).

This study included only adult participants aged between 19 and 80 years who exhibited venous reflux through Doppler ultrasound examination. The study defined reflux as reflux lasting at least 0.5 seconds in superficial veins (either great or small saphenous) or 1 second in deep veins (femoral or popliteal), with inclusion criteria requiring at least one affected vein. The screening process began only after participants completed a 4-week washout period to stop all medications affecting venous circulation, such as analgesics, steroids, and venoactive drugs. The exclusion criteria included patients who exhibited no symptoms from varicose veins, those with acute deep vein thrombosis, and individuals who had undergone varicose vein treatments, such as endovenous ablation or surgery, within the past year. Baseline data included information on demographics, pre-existing diseases, occupation, and Clinical-Etiological-Anatomical-Pathophysiological (CEAP) classification, as well as average daily standing time and compression stocking use.

### Randomization

Eligible participants were randomly assigned (1:1) to receive either VVSE medication or therapeutic lifestyle change (TLC) alone (control). Block randomization was performed using a computer-generated allocation sequence, managed by an independent programmer with no involvement in the trial. Physicians and research coordinators assigned participants via a web-based system; however, the study was not blinded.

### Interventions

Patients in the VVSE group were orally administered Entelon (VVSE 150 mg) twice daily for 12 weeks. Both groups were instructed to follow a TLC program as standard care, including guidance on avoiding prolonged sitting or standing and elevating the legs, when possible, to improve venous return. Participants were encouraged to wear compression stockings throughout the 12-week follow-up, regardless of group assignment.

### Outcome assessments

Follow-up visits were conducted at 6 and 12 weeks. During each visit, participants received Doppler ultrasonography assessments to determine venous reflux times while they completed symptom evaluations through the Venous Clinical Severity Score (VCSS) and the Chronic Venous Insufficiency Quality of Life Questionnaire (CIVIQ-14).^21^^,^^22^

During the ultrasound examination, venous reflux was augmented by applying a rapid cuff inflation system (E20/AG101, Hokanson) 10 cm distal to the target site. The cuff was inflated to 100 mm Hg within 0.3 seconds, maintained at this pressure for 3 seconds, and then rapidly deflated (in <1 second). A GE ViVid IQ system assessed changes in blood flow direction, and reflux was confirmed if the reversed flow persisted for ≥0.5 seconds in superficial veins or ≥1 second in deep veins. All Doppler ultrasounds were performed by a blinded investigator who was an independent examiner blinded to the participants’ treatment allocations to ensure unbiased assessment of venous reflux measurements.

### Endpoints

The primary endpoint was the difference between mean venous reflux times at baseline and at 12 weeks for each patient. Secondary endpoints included: (1) changes in venous reflux times across individual veins; (2) proportion of patients achieving improved venous reflux; and (3) changes in VCSS and CIVIQ-14 scores. An improvement in venous reflux was defined as at least one varicose vein showing follow-up reflux times of 0.5 seconds or less for superficial veins, and 1 second or less for deep veins. Symptom improvement was assessed by comparing baseline scores with those at 6 and 12 weeks for both VCSS and CIVIQ-14.

### Statistical analyses

The study planned to include 200 patients, to compensate for an expected 20% dropout rate so that at least 160 participants would ultimately complete both the 12-week intervention and Doppler assessments. The estimate utilized venous reflux time data and applied a two-sided *t*-test (α = .05), which achieved ≥80% power to identify a 12.5% difference in venous reflux time alterations from baseline between the two study groups.^23^

All analyses were conducted on the modified intention-to-treat population, comprising all randomized patients who received study treatment and had at least one follow-up assessment, excluding those who withdrew consent. This study compared baseline characteristics, including demographic factors, comorbidities, occupation, average daily standing time, and compression stocking use, between groups by applying Student’s t-test or Wilcoxon rank-sum test to continuous variables and χ^2^ test or Fisher’s exact test to categorical variables. The study used a mixed model for repeated measures to evaluate venous reflux time variations from baseline to 12 weeks. The analysis of covariance incorporated baseline reflux time adjustments to manage within-patient temporal changes. The χ^2^ test was applied to compare differences in the proportion of patients who achieved improved venous reflux. The effect size was quantified through calculations of absolute risk reduction, number needed to treat, and odds ratio with 95% confidence intervals (CIs). Pearson correlation analysis was performed to examine the relationship between changes in venous reflux time and symptomatic improvements (VCSS and CIVIQ-14 scores). Statistical significance was achieved when two-sided *P*-values were less than .05. Researchers performed all analyses with SAS software version 9.4 released by SAS Institute Inc.

## Results

### Patient characteristics

**Fig 1:**
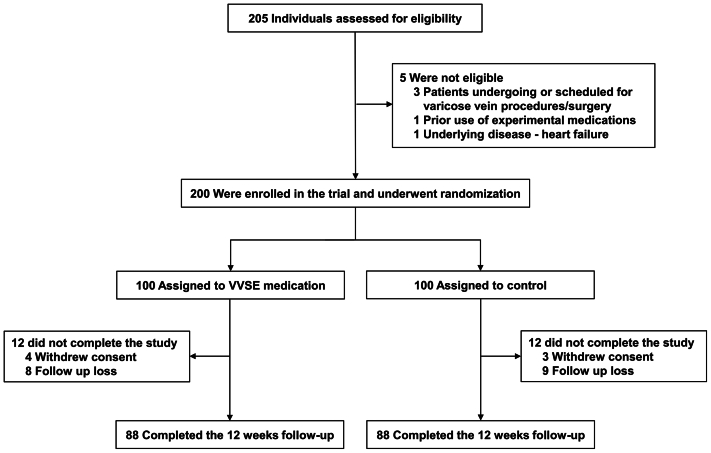
Flow diagram of the study. *VVSE*, *Vitis vinifera* seed extract.

Of 205 patients screened for eligibility (Fig 1), 200 met the inclusion criteria and were randomized in a 1:1 ratio to either the VVSE group or the control group, with each group containing 100 patients. Overall, 176 patients (88% of those randomized) completed the 12-week follow-up, including 88 each in the VVSE and control groups.

The study examined 176 participants, including 43 males (24.4%) and 133 females (75.6%), with an average age of 43.4 years (standard deviation, 15.5 years), as shown in Table I. The baseline demographic and clinical variables, including height, weight, and body mass index (BMI), showed no significant differences between groups. The rates of hypertension, diabetes, and dyslipidemia were similar across both the VVSE and control groups. There were no significant differences in CEAP classification distributions between the groups as shown by a *P*-value of .307. Patients from both the VVSE and control groups spent similar average daily standing hours (7.6 ± 3.8 vs 8.1 ± 3.7 hours; *P* = .454), and over 95% used compression stockings.

**Table I:** Baseline characteristics

	VVSE group (n = 88)	Control group (n = 88)	*P* value
Demographics			
Age, years	45.5 ± 15.7	41.4 ± 15.1	.083
Female	70 (79.5)	73 (82.9)	.562
Height, cm	161.7 ± 7.3	163.1 ± 6.6	.163
Weight, kg	62.4 ± 12.2	60.9 ± 12.2	.333
BMI, kg/m^2^	23.7 ± 3.6	22.8 ± 3.7	.067
Current smoker	5 (5.7)	5 (5.7)	1.000
Pre-existing disease			
Hypertension	15 (17.1)	14 (15.9)	.839
Diabetes	6 (6.8)	5 (5.7)	.755
Dyslipidemia	20 (22.7)	12 (13.6)	.118
Occupation			.070
Health care professional	36 (40.9)	48 (54.6)	
Office workers	18 (20.5)	6 (6.8)	
Manufacturing workers	1 (1.1)	4 (4.6)	
Sales and service workers	11 (12.5)	13 (14.8)	
Others	22 (25.0)	17 (19.3)	
CEAP classification			.307
C2	71 (71.6)	63 (80.7)	
C3	10 (19.3)	17 (11.4)	
C4	7 (9.1)	8 (8.0)	
Lifestyle and behavioral factors			
Average daily standing time, h	7.6 ± 3.8	8.1 ± 3.7	.454
Compression stocking use	85 (96.6)	87 (98.9)	.621

*BMI,* Body mass index; *CEAP,* clinical, etiological, anatomical, and pathophysiological classification; *VVSE, Vitis vinifera* seed extract.

Data are presented as the mean ± standard deviation or number (%).

### Improvements in venous reflux time

**Fig 2:**
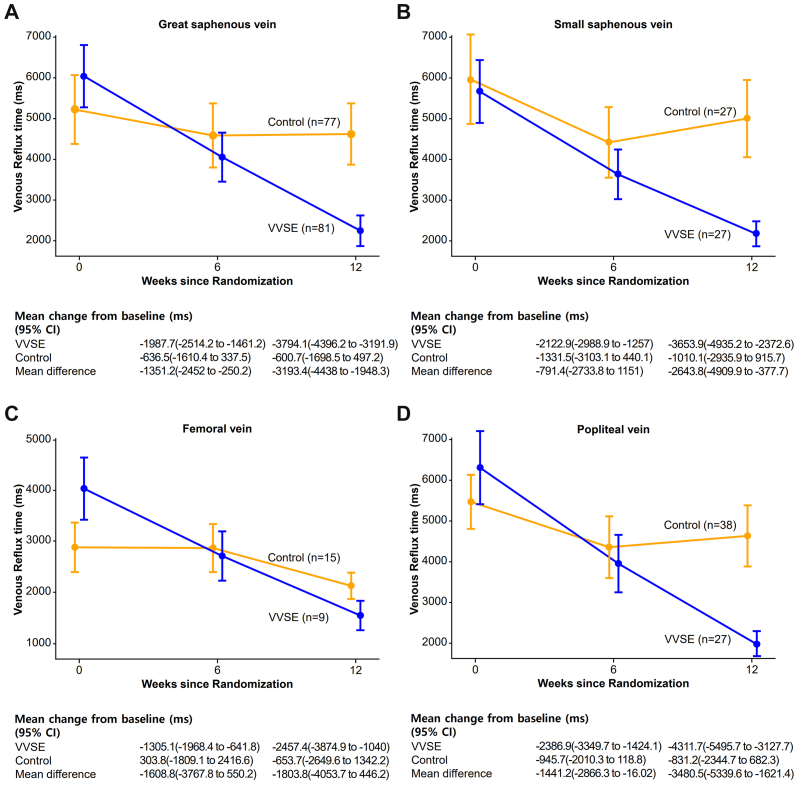
Changes in venous reflux time via vein segment at baseline, 6, and 12 weeks between the *Vitis vinifera* seed extract (*VVSE*) and control groups mean venous reflux time changes from baseline to 12 weeks for **(A)** great saphenous vein, **(B)** small saphenous vein, **(C)** femoral vein, and **(D)** popliteal vein. *Blue lines* represent VVSE group; *orange lines* represent control group. Error bars indicate 95% confidence intervals (*CIs*). Numbers in parentheses represent the total number of affected veins examined in each group. Mean changes from baseline with 95% CIs and mean differences between groups are shown below each panel.

Overall, VVSE patients demonstrated significantly greater reductions in mean venous reflux time compared with controls (Table II). The initial measurement showed mean venous reflux times per patient of 5983 ± 3137 ms and 5152 ± 3621 ms for the VVSE and control groups, respectively (*P* = .106). The VVSE group experienced an adjusted change from a baseline of −3599 ms (95% CI, −4077 to −3121 ms) compared with −1109 ms (95% CI, −1588 to −631 ms) in the control group, resulting in a treatment effect of −2490 ms (95% CI, −3168 to −1811 ms) with statistical significance (*P* < .001). Both superficial and deep veins showed significant decreases in reflux time with VVSE treatment, with great saphenous vein reflux time decreasing nearly four times more than in controls. The baseline venous reflux time for the great saphenous vein was 6037 ± 3659 ms and 5218 ± 4046 ms for the VVSE and control groups, respectively. The adjusted reflux time change at 12 weeks was −3497 ms (95% CI, −4082 to −2913 ms) for the VVSE group, whereas the control group showed a change of −912 ms (95% CI, −1512 to −313 ms), resulting in a treatment effect of −2585 ms (95% CI, −3424 to −1746 ms) with statistical significance (*P* < .001) (Fig 2). The small saphenous vein showed mean reflux time at randomization of 5624 ± 3867 ms in the VVSE group and 5939 ± 5491 ms in the control group, changing to −3749 ms (95% CI, −4912 to −2586 ms) and −915 ms (95% CI, −2078 to 248 ms) after 12 weeks, respectively, yielding a treatment effect of −2834 ms (95% CI, −4479 to −1189; *P* = .001).

**Table II:** Venous reflux time at randomization and 12 weeks

Variable	VVSE group (n = 88)	Control group (n= 88)	*P*	Treatment effect^a^ (95% CI)
Mean venous reflux time per patient				
At randomization	5983 ± 3137	5152 ± 3621	.106	
At 12 weeks	2114 ± 1513	4312 ± 3276	<.001	
Adjusted change at 12 weeks (95% CI)	−3599 (−4077 to −3121)	−1109 (−1588 to −631)	<.001	−2490 (−3168 to −1811)
Great saphenous vein reflux time				
Total number of veins examined	81	77		
At randomization	6037 ± 3659	5218 ± 4046	.184	
At 12 weeks	2243 ± 1801	4618 ± 3599	<.001	
Adjusted change at 12 weeks (95% CI)	−3497 (−4082 to −2913)	−912 (−1512 to −313)	<.001	−2585 (−3424 to −1746)
Small saphenous vein reflux time				
Total number of veins examined	27	27		
At randomization	5624 ± 3867	5939 ± 5491	.808	
At 12 weeks	1970 ± 1534	4929 ± 4743	.004	
Adjusted change at 12 weeks (95% CI)	−3749 (−4912 to 2586)	−915 (−2078 to 248)	.001	−2834 (−4479 to −1189)
Femoral vein reflux time				
Total number of veins examined	15	9		
At randomization	4009 ± 2885	2872 ± 2298	.327	
At 12 weeks	1552 ± 1365	2219 ± 1259	.111	
Adjusted change at 12 weeks (95% CI)	−2101 (−2795 to −1406)	−1248 (−2149 to −346)	.138	−852 (−2003 to −297)
Popliteal vein reflux time				
Total number of veins examined	38	27		
At randomization	6303 ± 4275	5468 ± 3154	.392	
At 12 weeks	1991 ± 1885	4637 ± 3570	.001	
Adjusted change at 12 weeks (95% CI)	−4064 (−4875 to −3254)	−1179 (−2142 to −216)	<.001	−2885 (−4148 to −1623)
Patients with improvement in venous reflux^b^				
No improvement	45/88 (51.1%)	67/88 (76.1%)		
Improvement	43/88 (48.9%)	21/88 (23.9%)	<.001	3.05 (1.61–5.81)

*CI,* Confidence interval; *VVSE, Vitis vinifera* seed extract.

Data are presented as mean ± standard deviation or number (%) unless otherwise indicated.

a The treatment effect is expressed as the mean difference with a 95% CI for all variables.

b Improvement in venous reflux was defined as a follow-up reflux time of ≤0.5 seconds for superficial veins and ≤1 second for deep veins in the affected varicose vein.

Notably, VVSE demonstrated significant efficacy in reducing deep vein reflux. For the deep vein, the popliteal vein showed a reflux time change of −4064 ms (95% CI, −4875 to −3254 ms) for the VVSE group, whereas the control group exhibited a change of −1179 ms (95% CI, −2142 to −216 ms), which resulted in a treatment effect of −2885 ms (95% CI, −4148 to −1623 ms) with statistical significance (*P* < .001).

### Treatment efficacy and symptom improvement

**Fig 3:**
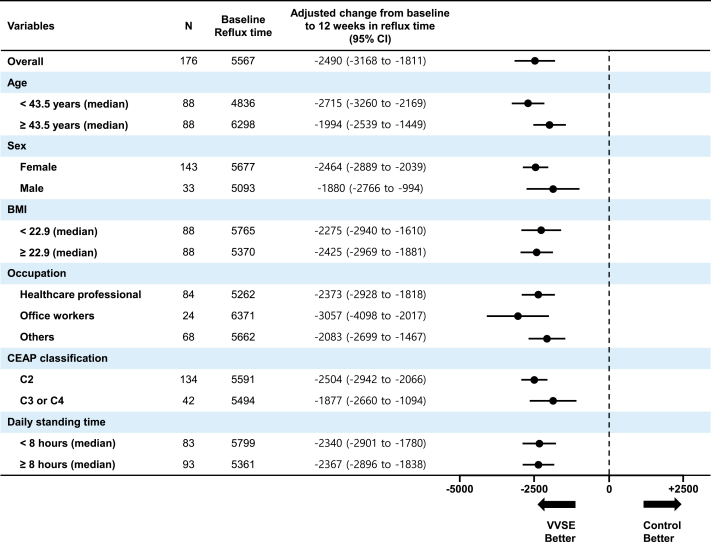
Estimated treatment effect by pre-specified subgroups forest plot showing the adjusted treatment effect (*Vitis vinifera* seed extract [VVSE] vs control) on venous reflux time reduction across prespecified subgroups. Values represent the mean difference in venous reflux time change from baseline to 12 weeks (ms) with 95% confidence intervals (*CIs*). All subgroups demonstrate consistent benefit favoring VVSE treatment. *BMI*, Body mass index; *CEAP*, Clinical-Etiological-Anatomical-Pathophysiological classification.

A significantly higher proportion of VVSE patients achieved improved venous reflux compared with controls. In the VVSE group, 43 of 88 patients (48.9%) reached improved venous reflux at 12 weeks compared with 21 of 88 patients (23.9%) in the control group, yielding an absolute risk reduction of 0.25 and an number needed to treat of 4 with an odds ratio of 3.05 (95% CI, 1.61-5.81; *P* < .001). The subgroup analysis revealed that the beneficial effects of VVSE maintained consistency across main baseline characteristics such as age, sex, BMI, occupation, and CEAP classification in Fig 3.

**Fig 4:**
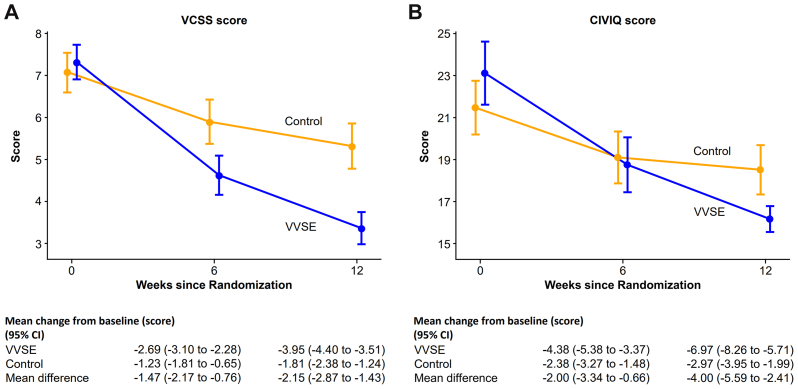
Changes in Venous Clinical Severity Score (*VCSS*) and Chronic Venous Insufficiency Quality of Life Questionnaire (*CIVIQ-14*) score between the *Vitis vinifera* seed extract (*VVSE*) and control groups at baseline and at 6 and 12 weeks. Changes in **(A)** VCSS and **(B)** CIVIQ-14 scores from baseline to 12 weeks. *Blue lines* represent VVSE group; *orange lines* represent control group. Error bars indicate 95% confidence intervals (*CIs*). Decreasing scores indicate symptom improvement and better quality of life. The VVSE group demonstrated significantly greater symptom relief compared with controls in both measures.

**Fig 5:**
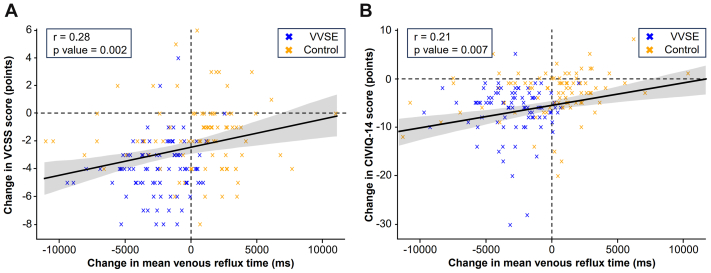
Correlation between changes in mean venous reflux time and patient-reported symptom scores. Scatter plots showing the correlation between changes in mean venous reflux time (x-axis) and improvements in **(A)** Venous Clinical Severity Score (*VCSS*) and **(B)** Chronic Venous Insufficiency Quality of Life Questionnaire (*CIVIQ-14*) scores from baseline to 12 weeks. *Blue crosses* represent *Vitis vinifera* seed extract (*VVSE*) group patients; *orange crosses* represent control group patients. Regression lines with 95% confidence intervals (CIs) are shown. Negative values on both axes indicate improvement.

The VVSE group showed significantly superior symptom improvement compared with the control group, according to both the VCSS and CIVIQ-14 measurements (Fig 4). The VCSS score decreased by −3.95 points (95% CI, −4.40 to −3.51 points) in participants receiving VVSE compared with −1.81 points (95% CI, −2.38 to −1.24 points) in the control group, resulting in a mean difference of −2.15 (95% CI, −2.87 to −1.43; *P* < .001). Furthermore, the CIVIQ-14 score showed an improvement of −6.97 points (95% CI, −8.26 to −5.71 points) for the VVSE group vs −2.97 points (95% CI, −3.95 to −1.99 points) in the control group, which produced a mean difference of −4.0 (95% CI, −5.59 to −2.41; *P* < .001). Correlation analysis demonstrated a significant relationship between venous reflux time improvement and symptomatic relief, with moderate positive correlations observed for both VCSS (r = 0.28; *P* = .002) and CIVIQ-14 (r = 0.21; *P* = .007) score improvements (Fig 5).

## Discussion

### Hemodynamic and clinical benefits of VVSE

Overall, the present study demonstrated that VVSE significantly reduced venous reflux time among patients with lower extremity varicose veins when compared with those with TLC alone. This improvement was especially pronounced in the superficial venous system, particularly in the great and small saphenous veins. A substantial proportion of patients treated with VVSE achieved improved reflux times, representing one of the first studies to demonstrate objective improvement in venous hemodynamics with VVSE treatment. These hemodynamic improvements were supported by parallel enhancements in patient-reported outcomes. VCSS and CIVIQ-14 scores markedly improved in the VVSE group compared with controls, linking venous reflux reduction to symptom relief. This correlation between objective and subjective measures supports VVSE as a viable noninvasive treatment option, particularly for patients who may not be candidates for invasive procedures, or those who prefer to avoid them. Subgroup analyses revealed consistent benefits across age, sex, BMI, and CEAP classification. This broad effectiveness profile indicates potential utility across diverse patient populations and different stages of venous disease. The favorable response across CEAP classifications indicates that VVSE may be beneficial in both early and more advanced stages of venous insufficiency.

### Mechanism of action and therapeutic properties of VVSE

The therapeutic benefits of VVSE likely stem from its high proanthocyanidin content, which has capillary-stabilizing properties and reduced vascular permeability.^24^ Proanthocyanidins also exhibit antioxidant properties that may mitigate local inflammation and oxidative stress in venous insufficiency.^25^^,^^26^ VVSE may strengthen venous walls by preserving endothelial integrity and inhibiting collagen- and elastin-degrading enzymes, by reducing reflux.^27^ Additionally, these compounds may complement mechanical interventions such as compression stockings, potentially enhancing overall treatment outcomes through different therapeutic mechanisms.^28^

### Clinical implications and impact on treatment guidelines

Our findings are significant in the context of current treatment guidelines. To date, venoactive pharmacologic agents, including VVSE and related flavonoid compounds, have been assigned a class IIb recommendation for symptomatic patients with varicose veins (CEAP C2) in the updated United States guidelines, indicating moderate-quality evidence with uncertain benefit for our target population.^29^ A key uncertainty has been whether addressing structural insufficiency effectively improves functional deficits in venous insufficiency.^30^ This study addresses that gap via comprehensive integration of objective Doppler assessments with validated patient-reported measures, providing robust evidence for both physiological and symptomatic improvements.

VVSE significantly improved popliteal vein reflux time, which is clinically important as deep veins are not amenable to surgical intervention or ablation, leaving limited treatment options for patients with deep venous insufficiency.^31^^,^^32^ The improvement in deep venous hemodynamics supports VVSE as a valuable therapeutic option in such challenging cases.^33^ This finding offers a potential solution for those with deep venous reflux, who traditionally have few evidence-based treatment alternatives beyond compression therapy. Noninvasive pharmacological improvement of deep venous function could shift the management paradigm for deep venous insufficiency, particularly for patients with mixed superficial and deep venous disease.

### Limitations and future perspectives

This study had some limitations. First, the open-label design may introduce bias in subjective outcomes, although the blinded ultrasound assessments and objective Doppler measures minimize this risk. Second, the single-center study design limits the external validity of the results, and longer follow-up intervals are needed to assess the durability of the observed benefits. Third, adherence to compression stockings and lifestyle guidance, although emphasized, was not strictly monitored and may vary in real-world practice. Fourth, future studies could employ a crossover “mixing experiment” design, switching treatments between groups after 12 weeks to demonstrate both therapeutic efficacy and reversibility, providing stronger pharmacological proof of drug effect. Future multi-center, placebo-controlled trials could help identify patient subgroups that benefit most from VVSE.

## Conclusions

VVSE demonstrated significantly reduced venous reflux time across both superficial and deep venous systems in patients with varicose veins, while improving patient-reported symptoms. The beneficial effects were consistent across diverse patient subgroups and were particularly noteworthy in deep venous insufficiency, where treatment options are traditionally limited. Supported by objective hemodynamic and validated clinical assessments, VVSE may be a valuable noninvasive therapeutic option for comprehensive management of varicose veins.

## Author contributions

Conception and design: SB, NS, IJ

Analysis and interpretation: SB, NS

Data collection: SB, HK, MK, SP, IJ

Writing the article: SB, MK, IJ

Critical revision of the article: SB, HK, NS, MK, SP, IJ

Final approval of the article: SB, HK, NS, MK, SP, IJ

Statistical analysis: NS

Obtained funding: IJ

Overall responsibility: IJ

SB and SP contributed equally to this article and share co-first authorship.

## Funding

This study was supported by Hanlim Pharmaceuticals. Hanlim Pharmaceuticals had no involvement in the study design or collection, analysis, and interpretation of data.

## Disclosures

None.
