# Supplementary material for: Vitis vinifera seed extract reduces venous reflux time in patients with varicose veins: VICTORY randomized controlled trial
Source: J Vasc Surg Venous Lymphat Disord. 2025 Nov 15;14(1):102355. doi: 10.1016/j.jvsv.2025.102355 (PMC12741370; doi:10.1016/j.jvsv.2025.102355)
Supplement: Supplementary Material [file mmc1.pdf]

A Prospective, Open-label, Pragmatic Randomized Controlled Trial to Demonstrate the Effect of Improving the Venous Reflux Time by Vitis Vinifera Seed Extract in Patients with Varicose Vein of Lower Extremity:

VICTORY trial

Trial Protocol

Version No: 1.1.0

Document Date: October 1, 2023

NCT05851183

VICTORY TRIAL

Table of Contents

1. Research summary 3
2. Introduction 9
3. Objectives and hypothesis 10
4. Research protocol and trial design 12
5. Study population 14
6. Eligiblity criteria, sample size calculation 14
7. Research Procedures and Evaluation 16
8. Methods 17
9. Standard of care for varicose vein 23
10. Termination and withdrawal criteria 24
11. Data Analysis & Statistical Considerations 25
12. Care for the safety of the participants 27
13. Funding Agency 33
14. Study schedule 33
15. Study period 33
16. References 34
17. Patient Consent Form 35

1. Research summary

**Title**	A Prospective, Open-label, Pragmatic Randomized Controlled Trial to Demonstrate the Effect of Improving the Venous Reflux Time by Vitis Vinifera Seed Extract in Patients with Varicose Vein of Lower Extremity (VICTORY trial)
**Study objective**	To evaluate the improvement in venous reflux time with Vitis Vinifera seed extract plus lifestyle versus lifestyle in patients with varicose vein of the lower extremities.
**Target population**	Patients with varicose vein of lower extremities
**Investigational medication**	Entelon® 150mg (Hanlim pharm.CO.LTD.)
**Principle investigator**	In Hyun Jung, M.D., PhD.<br>Professor, Department of Cardiology<br>Yongin Severance Hospital, Yonsei University College of Medicine
**Trial management**	SungA Bae, M.D., PhD<br>Assist Professor, Department of Cardiology<br>Yongin Severance Hospital, Yonsei University College of Medicine
**Study design**	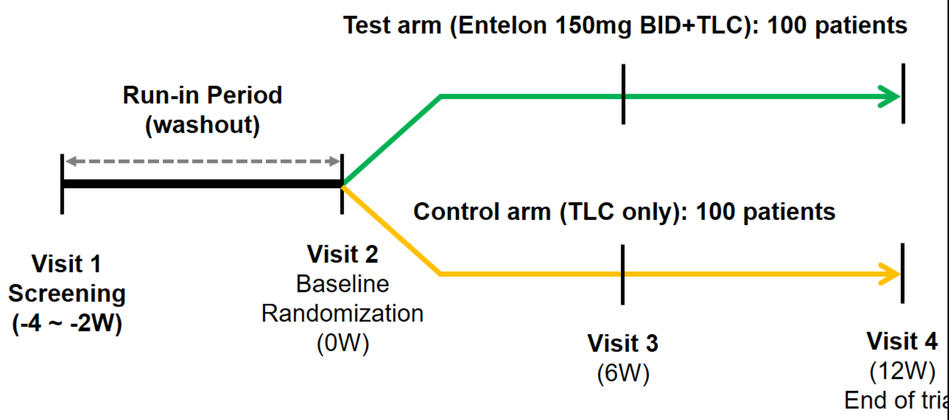<br><br>This study is designed as an investigator-initiated, single-center, open-label, pragmatic, randomized controlled trial. Patients with symptoms of lower extremity discomfort and leg edema and demonstrable venous reflux on lower extremity venous Doppler ultrasound (≥0.5 seconds in superficial veins or ≥1 second in deep veins) will be screened for inclusion in the study. All patients who meet the inclusion criteria and do not meet the exclusion criteria will be considered for inclusion.<br>Patients who are eligible for randomization will be randomized 1:1 to the therapeutic lifestyle change (TLC) only arm (control arm) and the medication (Entelon®) plus TLC arm (test arm). Patients will be treated for 12 weeks according to their assigned treatment arm. Patients will be evaluated for improvement in venous reflux in target vessels and

	symptomatic improvement by lower extremity venous Doppler ultrasonography at 6 and 12 weeks post-treatment follow-up.<br>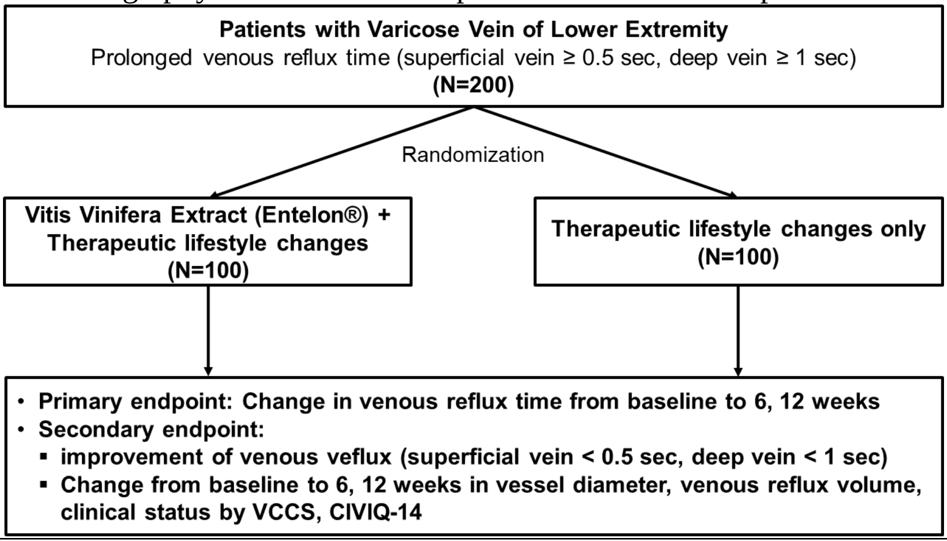
**Inclusion criteria**	1) Adult males and females aged between 19- and under 80-years-old<br>2) Patients with the following findings on venous Doppler ultrasound examination:<br>- Superficial veins (such as the great saphenous vein, small saphenous vein, etc.) showing venous reflux for 0.5 seconds or longer<br>- Deep veins (such as the femoral vein, etc.) showing venous reflux for 1 second or longer<br>3) Patients who have completed the washout period as described below by Visit 2, including the screening period:<br>- Analgesics, steroids, anti-inflammatory drugs, and venoactive medications (such as Entelon®, etc.): at least 4 weeks<br>4) Patients who voluntarily provide written informed consent to participate in this clinical trial
**Exclusion criteria**	1) Peripheral arterial occlusive disease in the lower limbs<br>2) Asymptomatic lower extremity varicose veins<br>3) Acute deep vein thrombosis<br>4) Frequent lower limb pain due to neuropathy<br>5) Patients who have undergone or are scheduled for varicose vein procedures/surgeries (However, patients who have undergone a procedure or surgery more than 1 year prior to the screening date are eligible to participate.)<br>6) Patients diagnosed with systemic diseases causing edema or thrombosis, such as heart failure, endocrine diseases (hypothyroidism, Cushing's syndrome, uncontrolled diabetes), allergic reactions to medications, urticaria and angioedema,

	malabsorption and protein-calorie malnutrition, obstructive sleep apnea, or thrombophilia, as determined by the investigator<br>A. History of malignancy within the past 5 years, but the following cases are eligible for clinical trial participation:<br>B. Those who have a history within the past 5 years but maintain a cured state without recurrence or metastasis<br>C. Those who have completed treatment for their tumor and have been disease-free for at least 5 years from the screening date<br>7) Those who have passed at least 1 year since the screening date after complete resection of basal cell carcinoma/squamous cell carcinoma, radical resection of thyroid papillary cancer, or successful treatment of cervical intraepithelial neoplasia<br>8) Severe renal dysfunction (serum creatinine levels more than twice the normal upper limit of the institution) on the screening date<br>9) Severe liver dysfunction (ALT or AST levels more than three times the normal upper limit of the institution) on the screening date<br>10) Need to receive diuretics or contraindicated medications and therapies that may affect the results of this clinical trial during the study period (However, patients who have been taking antihypertensive medications (calcium channel blockers, beta-blockers, angiotensin-converting enzyme inhibitors, vasodilators, vasoconstrictors) at the same dosage for at least 4 weeks (28 days) before screening and will maintain the same dosage and administration during the study period are eligible to participate)<br>11) History of clinically significant psychiatric disorders or alcohol abuse<br>12) History of hypersensitivity reactions to the investigational drug or its ingredients<br>13) Those who have participated in or are scheduled to participate in other clinical trials (investigational drugs, medical devices, health functional foods) within 12 weeks of the screening date<br>14) Pregnant or breastfeeding women<br>15) Women of childbearing potential who plan to become pregnant during the clinical trial participation period<br>16) Individuals deemed unsuitable for participation in the clinical trial by the investigator
**Primary endpoint**	Change in venous reflux time [Time Frame: 6, 12 weeks]<br>- We will assess the changes in venous reflux time of target vessel using lower limb venous Doppler ultrasound examinations.

**Secondary endpoint**	1) Improvement of venous reflux [Time Frame: 6, 12 weeks]<br>- We will assess the improvement of venous reflux of target vessel (superficial vein < 0.5 seconds, deep vein < 1 seconds)<br>2) Change in varicose vein size area [Time Frame: 6, 12 weeks]<br>- We will assess the changes in varicose vein diameter of target vessel using lower limb venous Doppler ultrasound examinations.<br>3) Change in venous reflux volume [Time Frame: 6, 12 weeks]<br>- We will assess the changes in venous reflux volume of target vessel using the Venous Doppler Reflux VTI x varicose vein size area.<br>4) Change of extracellular water ratio [Time Frame: 6, 12 weeks]<br>- We will assess the changes in extracellular water (ECW) ratio (ECW/total body water) of target limb using body composition analyzer (ACCUNIQ BC720®).<br>5) Improvement in VCSS [Time Frame: 6, 12 weeks]<br>- The Venous Clinical Severity Score (VCSS) is a clinical measure that assesses the severity of chronic venous diseases. The score ranges from 0 (indicating no symptoms) to 30 (indicating severe venous disease). The scoring system takes into account various factors including pain; varicose veins; venous edema; skin pigmentation; inflammation; induration; and active ulcers and their duration, size, and number. A higher score on the VCSS indicates a worse outcome, implying a more severe state of venous disease.<br>6) Improvement of CIVIQ-14 [Time Frame: 6, 12 weeks]<br>- The Chronic Venous Insufficiency Quality of Life Questionnaire (CIVIQ-14) is a valuable tool designed to evaluate the impact of chronic venous insufficiency on a patient's quality of life. The 'improvement' in CIVIQ-14 refers to the enhancement in scores, indicating better management of symptoms, greater comfort, and overall wellbeing of the patient over the course of treatment or intervention.
**Sample size**	200 patients
**Sample size calculation**	This study is an exploratory study to determine the effectiveness of venous reflux improvement after medication in patients with varicose veins, and there are limited prior studies to draw upon. Therefore, the study design is subject to a number of assumptions. In addition, it is not advisable to apply the same process for calculating the number of patients to meet the mathematical basis for a confirmatory study as for a confirmatory study when controlling for type 1 and type 2 errors.<br>Nevertheless, a few assumptions can be made to develop a power calculation process that is similar to that of a confirmatory study. According to the literature, the duration of reflux was measured slightly differently at each point, and we would like to calculate the number of

patients based on GSV, prox, which had the largest number of patients (Reference: J Vasc Surg. 2004 Aug;40(2):303-10.). In this study, we would like to conduct an analysis on the difference between the means, but no relevant evidence was found; therefore, we borrowed the median value of the references as the mean. This assumes that the number of n is large enough (174) according to the principle of central limit theorem, such that the gap between the median and the mean is not large. We also calculated the standard deviation using the range and the number of n. By calculating the standard deviation of other parameters together, we found that the standard deviation of the valve-carrying segments can be assumed as approximately 1.8 overall. The results of the drug group are expected to improve by about 10% to 15%, and we decided to conservatively assume an improvement of 12.5% to calculate the number of patients.

Valve-carrying segment	n	Median (s)	Range (s)
GSV, prox	174	6.1	0.7-10.0
GSV, dist	84	3.5	0.7-10.0
SSV	91	4.6	0.6-10.0

In this case, assuming a normal distribution and based on a Z-test with the same variance in both groups, the following calculation result was obtained under α=0.025, β=0.2. The calculation was performed using PASS15. A one-tailed test will be performed to determine superiority if the difference between the drug and lifestyle treatment groups is less than zero. Assuming that patients may drop out during the study, the dropout rate was set at 20% to account for dropout in a typical clinical setting.
Therefore, the total number of patients was calculated to be 160, with 80 patients in each group, and considering the dropout rate of 20%, we plan to recruit 200 patients, 100 in each group, to conduct the study.

Two-Sample Z-Tests Assuming Equal Variance

Numeric Results for Two-Sample Z-Test Assuming Equal Variance
Alternative Hypothesis: H1: δ = μ1 - μ2 < 0

Target Power	Actual Power	N1	N2	N	μ1	μ2	δ	σ	Alpha
0.80	0.80260	80	80	160	5.3	6.1	-0.8	1.8	0.025

Summary Statements
Group sample sizes of 80 and 80 achieve 80.260% power to reject the null hypothesis of equal means when the population mean difference is μ1 - μ2 = 5.3 - 6.1 = -0.8 with a standard deviation for both groups of 1.8 and with a significance level (alpha) of 0.025 using a one-sided two-sample equal-variance z-test.

Dropout-Inflated Sample Size

Dropout Rate	Sample Size			Dropout-Inflated Enrollment Sample Size		
	N1	N2	N	N1'	N2'	N'
20%	80	80	160	100	100	200

**Study period**	2023.07.01 ~ 2024.12.31<br>Patient enrollment: IRB approval date ~2024.04.31<br>End of follow-up period: 2024.07.31<br>Analysis and report: ~2024.12.31
**Funding Agency**	Hanlim pharm.CO.LTD.

2. Introduction

Varicose veins arise from a malfunction in the valves within the veins of the legs, which allows blood to be transported to the heart to flow backward into the lower extremities. This backflow results in pooling of blood in these areas, which subsequently triggers various symptoms. Typically, malfunction occurs in valves that are located in superficial veins, such as the great saphenous vein, which drains into the deep vein at the groin, the small saphenous vein at the calf, or perforating veins. This malfunction allows blood reflux, causing the veins in the calf to dilate. The primary cause is incompetence of the leg vein valves.

According to an analysis of varicose vein treatment data from 2016 to 2020, the number of treated patients increased by 50,000 from 162,000 in 2016 to 212,000 in 2020. This represents an average annual increase of 7%. With an aging population, the number of patients with varicose veins is expected to continue to rise. Varicose veins frequently occur and worsen in older individuals, women, those who have given birth, obese individuals, and those with jobs that require long periods of standing. If left untreated, varicose veins resulting from valve incompetence can worsen, leading to complications, such as skin pigmentation, swelling, and leg ulcers. Therefore, treatment is essential, particularly for patients with risk factors, and prevention is of paramount importance.

Patients typically visit clinics because of enlarged veins in the calf. However, various symptoms can occur when accompanied by valve incompetence. These include aches, spasms, fatigue, dull pain, night pain, heat sensations, and feelings of heaviness in the legs. It is also possible that musculoskeletal, neurological, or arterial issues trigger these symptoms, necessitating specialist consultations. Valve incompetence is crucial in cases of suspected varicose veins. Typically, Doppler ultrasonography is used to confirm reflux in the superficial veins (great and small saphenous veins) and deep veins (popliteal veins). If dilation of the branch veins is noted, a diagnosis is made and the treatment approach is determined. Even if symptoms are present in only one leg, an ultrasound examination is conducted on both sides.

Treatment of varicose veins can generally be divided into conservative and invasive procedures. Conservative treatment involves lifestyle modifications and the use of compression stockings. Surgical treatment may be required if these measures are ineffective or if they interfere with daily life. By removing or ablating the refluxing superficial veins using high-frequency radio waves or lasers, blood flow can be stopped, thereby relieving the symptoms. However, most of these treatments are not covered by insurance and can be relatively expensive, potentially

placing a significant financial burden on patients. Furthermore, because deep veins are responsible for 90% of blood transport, surgical treatment may not be possible, leaving only conservative treatment as an option.

Entelon®, a pharmaceutical that is derived from Vitis vinifera seeds, is used for non-invasive treatment of various conditions. The primary goal of this treatment is to improve symptoms related to veno-lymphatic insufficiency, such as feelings of heaviness in the legs, pain, and restless legs syndrome. Enteron is composed of Vitis vinifera seed (grape seed) extract, which is a natural compound. It strengthens the vascular wall by protecting collagen and elastin, improving their structure, and increasing fibronectin and elastonectin, which promote extracellular matrix binding. It also selectively binds to GAGs, promotes their synthesis, and inhibits their breakdown and trypsin activity. Additionally, grape seed extract protects vascular wall components (antioxidant action of Vit.C 50 times, Vit.E 20 times) and inhibits cholesterol accumulation. Since its launch in 1998, Enteron® has been used to treat symptoms such as heaviness, pain, and discomfort in the legs, lymphedema resulting from breast cancer treatment, and non-proliferative diabetic retinopathy. Its safety has been established since its release, and it has been shown to reduce leg volume compared to placebo in patients with leg swelling (Sano A, Tokutake S, Seo A. Proanthocyanidin-rich grape seed extract reduces leg swelling in healthy women during prolonged sitting. J Sci Food Agric. 2013 Feb;93(3):457-62.). However, the relationship between the degree of venous reflux and associated symptoms in patients with varicose veins is not well understood. Furthermore, there is a lack of research on whether the administration of Entelon® improves venous reflux in patients with varicose veins.

In this study, we aim to confirm the improvement of venous reflux through follow-up Doppler ultrasound examinations in patients with varicose veins who have been prescribed Enteron® along with therapeutic lifestyle changes.

3. Objectives and hypothesis

3.1. Objectives

To evaluate the improvement in venous reflux following Vitis vinifera seed extract (Enteron®) administration in patients with varicose veins of the lower extremities.

3.2. Hypothesis

We hypothesize that, among patients with vericose veins, the severity of symptoms is likely to

escalate in correlation with the increasing severity of venous reflux. We posit that the medication of Vitis Vinifera seed extract (Enteron®) will contribute to the improvement of venous reflux, subsequently leading to a reduction in symptoms.

3.3. Primary endpoint

Change in venous reflux time [Time Frame: 6, 12 weeks]: We will assess the changes in venous reflux time of target vessel using lower-limb venous Doppler ultrasound examinations.

3.4. Secondary endpoint

1) Improvement of venous reflux [Time Frame: 6, 12 weeks]

- We will assess the improvement of venous reflux of target vessel (superficial vein < 0.5 seconds, deep vein < 1 seconds)

2) Change in varicose vein size area [Time Frame: 6, 12 weeks]

- We will assess changes in varicose vein diameters of target vessel using lower-limb venous Doppler ultrasound examinations.

3) Change in venous reflux volume [Time Frame: 6, 12 weeks]

- We will assess the changes in venous reflux volume of target vessel using Venous Doppler Reflux VTI × varicose vein size area.

4) Change of extracellular water ratio of target limb [Time Frame: 6, 12 weeks]

- We will assess the changes in extracellular water (ECW) ratio (ECW/total body water) of target limb using body composition analyzer (ACCUNIQ BC720®).

5) Improvement in VCSS [Time Frame: 6, 12 weeks]

- The Venous Clinical Severity Score (VCSS) assesses the severity of chronic venous diseases. The score ranges from 0 (no symptoms) to 30 (severe venous disease). The scoring system considers various factors, including pain; varicose veins; venous edema; skin pigmentation; inflammation; induration; and active ulcers and their duration, size, and number. A higher VCSS indicates a worse outcome, implying a more severe state of venous disease.

6) Improvement of CIVIQ-14 [Time Frame: 6, 12 weeks]

- The Chronic Venous Insufficiency Quality of Life Questionnaire (CIVIQ-14) is a valuable tool designed to evaluate the impact of chronic venous insufficiency on quality of life. Improvement in the CIVIQ-14 refers to the enhancement in scores, indicating better management of symptoms, greater comfort, and overall well-being of the patient over the course of treatment or intervention.

4. Research protocol and trial design

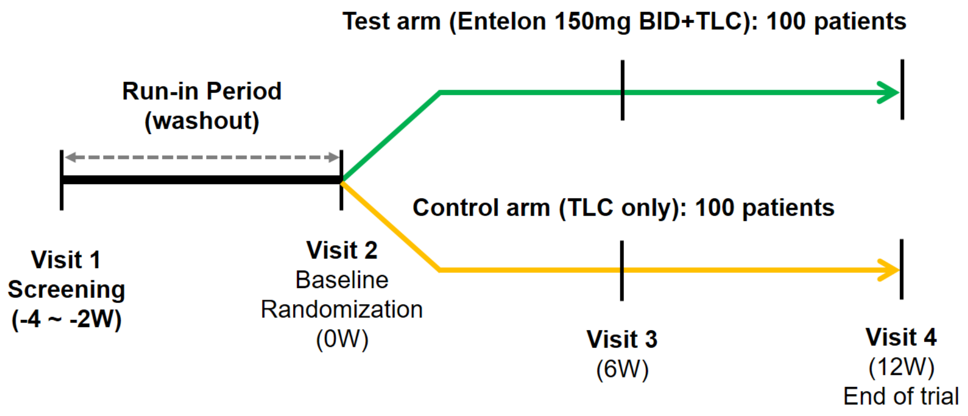

This study is designed as an investigator-initiated, single-center, open-label, pragmatic, randomized controlled trial. Patients with symptoms of lower extremity discomfort and leg edema and demonstrable venous reflux on lower extremity venous Doppler ultrasound (≥0.5 seconds in superficial veins or ≥1 s in deep veins) will be screened for inclusion in the study. All patients who meet the inclusion criteria will be considered for inclusion.

Patients who are eligible for randomization will be randomized 1:1 to the therapeutic lifestyle change (TLC) only arm (control arm) or the medication (Entelon®) plus TLC arm (test arm). The patients will be treated for 12 weeks according to their assigned treatment arms. Patients will be evaluated for improvement in venous reflux in the target vessels and symptomatic improvement using lower-extremity venous Doppler ultrasonography at 6 and 12 weeks post-treatment.

4.1. Randomization

Patients will be randomized to either the medication + TLC group or the TLC only group at the

time of enrollment in a 1:1 ratio. We plan to conduct block randomization. This process will be executed using a web-based randomization program that is managed by an independent organization.

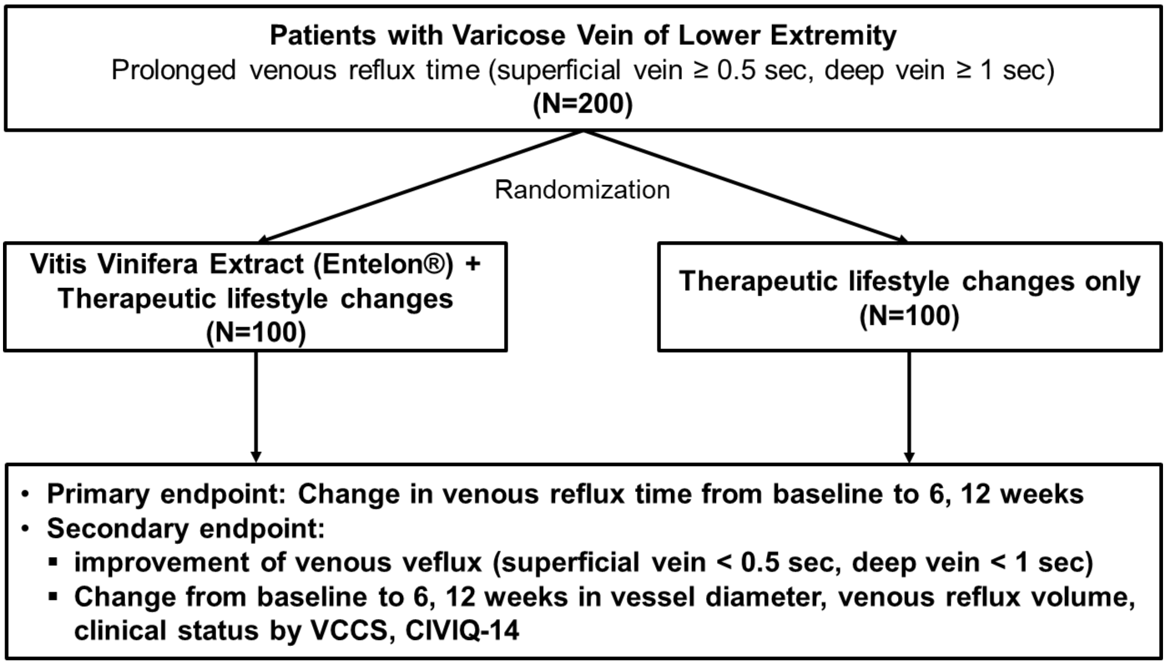

4.2. Blinding assignment

Patients will be openly assigned without blinding for the conduct of the research.

4.3. Therapeutic lifestyle changes group

Implementing TLCs is a critical component of a comprehensive treatment plan for varicose veins. These non-invasive interventions focus on amending daily habits and behaviors that contribute to the progression of the condition and exacerbation of symptoms. A key aspect of TLCs is the optimization of postural habits. Patients are advised to avoid extended sitting or standing periods, which exert additional pressure on the venous system in the lower extremities and exacerbate venous insufficiency. During rest periods, elevating the legs closer to the heart is recommended because this simple yet effective adjustment promotes venous return and reduces the gravitational burden on the venous valves of the legs. The use of graduated compression stockings during waking hours is another therapeutic lifestyle intervention that is often prescribed for patients with varicose veins. These stockings exert the highest amount of pressure on the ankle, with the pressure gradually decreasing up the leg, thereby mimicking the

physiological pumping function of the calf muscles. This supports the veins in pushing blood back towards the heart, improving overall blood circulation and reducing leg swelling and discomfort associated with varicose veins.

Additional TLCs include maintaining optimal body weight and engaging in regular physical exercise. Being overweight or obese can increase intra-abdominal pressure, which can impose an additional burden on the leg veins. Regular physical activity, specifically exercises that engage the calf muscles, can improve muscle tone, enhance the efficiency of the calf muscle pump, and consequently, improve venous return. Walking or cycling are often recommended because of their effectiveness in promoting lower limb venous return.

4.4. Medication + TLC group

Patients selected for the combined approach of pharmacotherapy and lifestyle modifications will receive Entelon® 150 mg twice daily for a duration of 12 weeks. This pharmacological intervention should be complemented by the adoption of improved lifestyle habits, including wearing compression stockings.

5. Study population

Patients with varicose veins of lower extremities will be enrolled.

6. Eligiblity criteria, sample size calculation

6.1. Eligibility criteria

6.1.1. Inclusion criteria

1) Adult males and females aged between 19- and under 80-years-old
2) Patients with the following findings on venous Doppler ultrasound examination:
   - Superficial veins (such as the great and small saphenous veins) showing venous reflux of 0.5 seconds or longer
   - Deep veins (such as the femoral vein) showing venous reflux for 1 second or longer
3) Patients who have completed the washout period as described below by Visit 2, including the screening period:

- Analgesics, steroids, anti-inflammatory drugs, and venoactive medications (such as Entelon®, etc.): at least 4 weeks

4) Patients who voluntarily provide written informed consent to participate in this clinical trial

6.1.2. Exclusion criteria

1) Peripheral arterial occlusive disease in the lower limbs
2) Asymptomatic lower extremity varicose veins
3) Acute deep vein thrombosis
4) Frequent lower limb pain due to neuropathy
5) Patients who have undergone or are scheduled for varicose vein procedures/surgeries (However, patients who have undergone a procedure or surgery more than 1 year prior to the screening date are eligible to participate.)
6) Patients diagnosed with systemic diseases causing edema or thrombosis, such as heart failure, endocrine diseases (hypothyroidism, Cushing's syndrome, and uncontrolled diabetes), allergic reactions to medications, urticaria and angioedema, malabsorption, protein-calorie malnutrition, obstructive sleep apnea, or thrombophilia, as determined by the investigator
    A. History of malignancy within the past 5 years, but the following cases are eligible for clinical trial participation:
    B. Those who have a history within the past 5 years but maintain a cured state without recurrence or metastasis
    C. Those who have completed treatment for their tumors and have been disease-free for at least 5 years from the screening date
7) Those who have passed at least 1 year since the screening date after complete resection of basal cell carcinoma/squamous cell carcinoma, radical resection of thyroid papillary cancer, or successful treatment of cervical intraepithelial neoplasia
8) Severe renal dysfunction (serum creatinine levels more than twice the normal upper limit of the institution) on the screening date
9) Severe liver dysfunction (ALT or AST levels more than three times the normal upper limit of the institution) on the screening date

10) Need to receive diuretics or contraindicated medications and therapies that may affect the results of this clinical trial during the study period (However, patients who have been taking antihypertensive medications (calcium channel blockers, beta-blockers, angiotensin-converting enzyme inhibitors, vasodilators, and vasoconstrictors) at the same dosage for at least 4 weeks (28 days) before screening and who will maintain the same dosage and administration during the study period are eligible to participate)
11) History of clinically significant psychiatric disorders or alcohol abuse
12) History of hypersensitivity reactions to the investigational drug or its ingredients
13) Those who have participated in or are scheduled to participate in other clinical trials (investigational drugs, medical devices, health functional foods) within 12 weeks of the screening date
14) Pregnant or breastfeeding women
15) Women of childbearing potential who plan to become pregnant during the clinical trial participation period
16) Individuals deemed unsuitable for participation in the clinical trial by the investigator

7. Research Procedures and Evaluation

7.1. Visit 1 (Screening Visit, Weeks 4 to 2 at randomization)

Participants will visit the study site within 2–4 weeks before the study to receive an explanation of the study, provide informed consent to participate, and undergo the screening tests described below.

Patients with clinically significant abnormalities at this time will be excluded from the study.

1) Assignment of screening numbers: Screening numbers will be assigned according to the order in which written consent is received
2) Review of patient medical history and demographic information
3) Review of inclusion/exclusion criteria eligibility
4) Measurement of vital signs, height, and weight
5) Assessment of existing medications
6) Performance of blood tests and urinalysis
7) Performance of lower extremity venous Doppler ultrasound imaging: Patients with confirmed venous reflux times of 0.5 seconds in the bilateral superficial veins and 1 second or more in the deep veins

8) Assessment of symptoms: Venous Clinical Severity Score (VCSS), CIVIQ-14

7.2. Visit 2 (Randomization Visit, Day 0)

1) Review of inclusion/exclusion criteria
2) Review of medical/Surgical History
3) Physical examination and measurement of vital signs
4) Randomization (assignment of registration number)

7.3. Visit 3 (Midterm Assessment Visit, 6 weeks from randomization visit)

1) Lower extremity venous Doppler ultrasound (required)
2) Assessment of symptoms: Venous Clinical Severity Score (VCSS), CIVIQ-14
3) Performance of blood tests, urinalysis (optional)
4) Concomitant medications check (antiplatelet, antithrombotic, antihyperlipidemic).
5) Review of lifestyle therapy - frequency of stocking wear, pharmacotherapy - medication adherence checks
6) Monitoring of adverse events

7.4. Visit 4 (End Visit, 12 weeks from randomization visit)

1) Lower extremity venous Doppler ultrasound (required)
2) Assessment of symptoms: Venous Clinical Severity Score (VCSS), CIVIQ-14
3) Performance of blood tests, urinalysis (optional)
4) Concomitant medications check (antiplatelet, antithrombotic, antihyperlipidemic).
5) Review of lifestyle therapy - frequency of stocking wear, pharmacotherapy - medication adherence checks
6) Monitoring of adverse events

8. Methods

8.1. Demographic Information

At Visit 1 (Screening Start Date), we will conduct a demographic survey of the participants' initials, date of birth, sex, age, smoking, alcohol use, BMI, occupation, and standing time.

8.2. Medical History and Prior/Concomitant Medications

Medical history is defined as any medical condition that has been ongoing for less than 1 year as of Visit 1 (screening start date), including past medical history (hypertension, diabetes, hyperlipidemia, previous PCI, previous myocardial infarction, peripheral vascular disease, heart failure, CVA, dialysis, CABG) and clinically significant diagnostic findings from blood chemistry, urinalysis, physical examination, endoscopy, etc. performed up to Visit 2. In addition, the history of medications within 4 weeks (28 days) of Visit 1 (screening start date) and up to Visit 2 will be investigated and collected as prior medications, and the history of medications at Visit 2 will be collected as concomitant medications.

8.3. Vital Signs and Physical Examination

At each visit, the sitting blood pressure (systolic and diastolic), heart rate, and body temperature will be measured. Blood pressure, heart rate, and temperature will be measured after 5 minutes of rest and, if possible, prior to all scheduled tests.

At each visit, the patients be weighed and observed for the following: Height will only be measured during screening. A physical examination will be performed by the investigator, who will examine the patient's body for clinically significant abnormal symptoms and signs, excluding medical history, by palpation, tapping, and auscultation. The physical examination that is performed after screening focuses on the presence of clinically significant changes compared to the previous examination. Physical examinations include the weight, cardiovascular system, peripheral vascular system, skin/mucous membranes, eyes (excluding vision impairment), ENT, respiratory system, musculoskeletal system (excluding hernias), infectious diseases (excluding childhood diseases), gastrointestinal tract/liver/biliary system (excluding appendectomy), endocrine system, renal/genitourinary system, neurological/psychiatric system, tumors, fractures, and surgery.

8.4. Blood and urine tests

Test results that are obtained within 28 days of Visit 1 (screening start date) at the same institution, even those that are obtained before consent to participate is obtained, may be used. However, if the substituted test has clinically significant abnormal results, blood and urine tests must be performed before Visit 2 to assess the patient’s suitability for study participation.

Tests may also be performed at Visits 3 and 4 but will be performed under the standard of care

and will be paid for by the patient. Elective tests that are performed during outpatient visits may be necessary:

- If the healthcare provider deems it necessary to assess the patient’s health status
- If the patient’s condition or symptoms change
- If, in the judgment of the healthcare provider, there are other times when blood and urine tests are necessary

Blood test list	
CBC test	Hb, Hct, platelet
Blood Chemistry Tests	total bilirubin, BUN, creatinine, Cystatin C, ALP, AST, ALT, CPK, total Protein, Albumin, Alkaline Phosphatase, Glucose, Lipid battery (TG, TC, HDL, LDL), CRP, HbA1c*
Urine test list	
Urinalysis	Protein, Glucose, Blood (Heme)

8.5. Lower extremity venous Doppler ultrasound examination

Doppler ultrasound examination utilizes the characteristic of increased frequency changes as blood flow velocity accelerates to observe frequency changes based on the blood flow direction. Measurements are taken using GE ViVid IQ equipment. When the blood flow is directed towards the probe, the Doppler signal frequency increases and is displayed above the baseline. Conversely, if the blood flow moves away from the probe, it is displayed below the baseline. The patients are positioned with the bed inclined at 60–90 °, and the presence or absence of venous reflux in each area is confirmed. If reflux is present, its velocity, duration, and quantity will be recorded. To induce proximal venous reflux, a rapid cuff inflation system (E20/AG101, Hokanson) with maximum pressure of 100 mm Hg was used to augment flow, positioning the cuff 10 cm below the target area. Time to inflation was 0.3 seconds, inflation was maintained for 1 second, and deflation was achieved in less than 1 second. If a standing posture is not feasible, measurements can be performed in the sitting or 'Reverse Trendelenburg' position. The vessels subject to examination are limited to the superficial veins (great and small saphenous veins) and deep veins (femoral and popliteal veins) of the lower extremities. Superficial veins are diagnosed as venous reflux if the sign of reflux time is visible for more than 0.5 seconds, and deep veins are diagnosed if the sign of reflux time is visible for more than 1 second. In addition to these procedures, the examination will be performed by an

independent assessor who is not aware of the group assignments to ensure unbiased assessment and measurement.

References:

- Gloviczki P, Lawrence PF, Wasan SM et al. The 2022 Society for Vascular Surgery, American Venous Forum, and American Vein and Lymphatic Society clinical practice guidelines for the management of varicose veins of the lower extremities. Part I. Duplex Scanning and Treatment of Superficial Truncal Reflux: Endorsed by the Society for Vascular Medicine and the International Union of Phlebology. J Vasc Surg Venous Lymphat Disord. 2023 Mar;11(2):231-261.
- Labropoulos N, Tiongson J, Pryor L, et al. Definition of venous reflux in lower-extremity veins. J Vasc Surg 2003;38:793-8.
- Labropoulos N, Leon LR Jr. Duplex evaluation of venous insufficiency. Semin Vasc Surg 2005;18:5-9.
- Marianne G. De Maeseneer European Society for Vascular Surgery (ESVS) 2022 Clinical Practice Guidelines on the Management of Chronic Venous Disease of the Lower Limbs CLINICAL PRACTICE GUIDELINE DOCUMENT Vol63. P184-267, FEBRUARY 01, 2022
- AIUM Practice Parameter for the Performance of a Peripheral Venous Ultrasound Examination. J Ultrasound Med. 2020 May;39(5):E49-E56. doi: 10.1002/jum.15263. Epub 2020 Mar 12. PMID: 32162338.

8.6. Rationale of calculation for venous reflux volume

In assessing venous reflux volume, our proposed methodology employs principles reminiscent of fluid dynamics. Drawing significant parallels with techniques that are used in echocardiography for stroke volume and valve regurgitation estimation, our approach utilizes the calculation of Venous Doppler Reflux Velocity Time Integral (VTI) multiplied by the cross-sectional area of the varicose vein (Zoghbi WA, et al. American Society of Echocardiography. Recommendations for evaluation of the severity of native valvular regurgitation with two-dimensional and Doppler echocardiography. J Am Soc Echocardiogr. 2003 Jul;16(7):777-802, Enriquez-Sarano M, et al. Quantitative Doppler assessment of valvular regurgitation. Circulation. 1993 Mar;87(3):841-8.).

$$\mathrm{SV} = \mathrm{CSA} \times \mathrm{VTI} = \pi \mathrm{d}^2/4 \times \mathrm{VTI} = 0.785\ \mathrm{d}^2 \times \mathrm{VTI}$$

The fundamental concept behind this calculation is that the total volume of reflux across a vein during the period of distal augmentation is a function of the reflux velocity and the cross-sectional area of the vein from which it originates. According to the principle of conservation of mass, a fundamental tenet in fluid dynamics, the volume flowing into a system must equate

to the volume flowing out, assuming no volume addition or subtraction (Acheson, D. J. (1990). Elementary Fluid Dynamics. Clarendon Press). As such, the product of the VTI and the vein's cross-sectional area gives us the total reflux volume during the period of distal augmentation. This methodology offers a measurable and repeatable means of quantifying the venous reflux volume, providing an objective way to gauge both the effectiveness of treatments and disease progression over time. The reliability of the method depends on the accuracy of both VTI and cross-sectional measurements, requiring meticulous techniques and consideration of variables such as pressure changes and vein distension in different body positions. Despite these considerations, with the standardization of the measurement technique and the control of confounding variables, this method could serve as a valuable tool for the study and treatment of venous insufficiency.

8.7. Measurement of extracellular water ratio using body composition analyzer

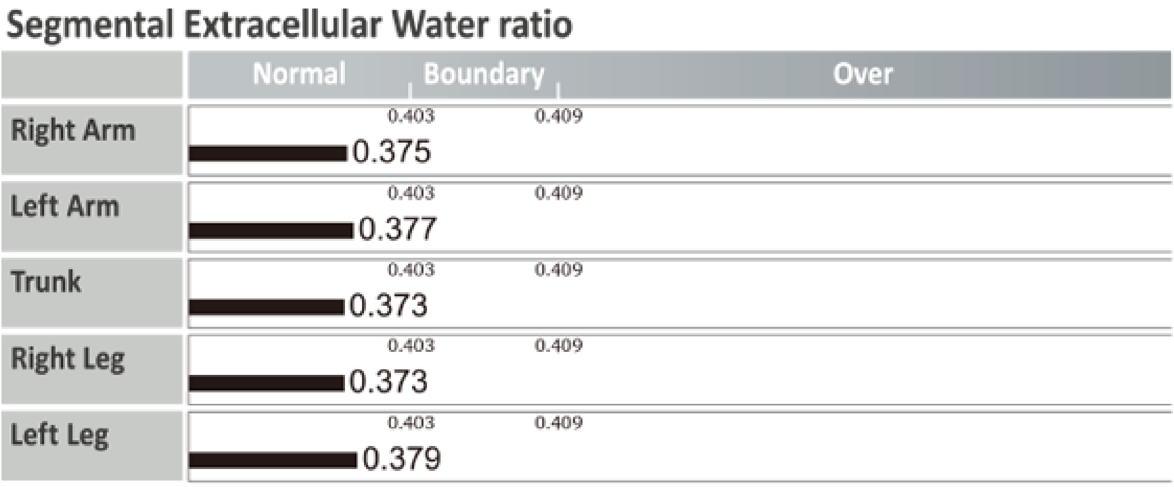

It provides the values of body water, intracellular water, extracellular water, and extracellular water ratio for each body part in separate result sheets in order to evaluate the balance of body water. About 50-60% of the human body is made up of water, which is further divided into intracellular water (about 2/3) and extracellular water (about 1/3). In body water evaluation, it is important to evaluate the degree of water balance through extracellular water ratio (extracellular water / body water), not simply water content. If body water is imbalanced, edema is suspected.

ACCUNIQ BC720 measures extracellular water through low frequency and total body water through high frequency, enabling evaluation of intracellular and extracellular water throughout

the human body.

8.8. Symptom Assessment: VCSS, CIVIQ-14

Patients who meet the inclusion criteria will be assessed pre- and post-study using the Venous Clinical Severity Score (VCSS) questionnaire and the Chronic Venous Insufficiency Quality of Life Questionnaire (CIVIQ-14).

1) VCSS (Venous Clinical Severity Score)

Attribute	Absent (0)	Mild (1)	Moderate(2)	Severe (3)	Score
Pain	None	Occasional	Daily	Daily with med.	
Varicose veins	None	Few	Multiple	Extensive	
Venous edema	None	Evening only	Afternoon	Morning	
Skin pigmentation	None	Limited, old	Diffuse, more recent	Wider, recent	
Inflammation	None	Mild cellulitis	Moderate cellulitis	Severe	
Induration	None	Focal <5 gaiter	<1/3 gaiter	>1/3 gaiter	
No. of active ulcers	None	1	2	>2	
Active ulcer size	None	<2cm	2-6cm	>6cm	
Ulcer duration	None	<3 months	3-12 months	>1 year	
Compression	None	Intermittent	Most days	Fully complaint	
				Total	

Reference: J Vasc Surg. 2011 Dec;54(6 Suppl):2S-9S.

2) CIVIQ-14 (Chronic Venous Insufficiency Quality of Life Questionnaire)

**1) During the past four weeks, have you had any pain in your ankles or legs, and how severe has this pain been?** *Circle the number that applies to you.*				
**No pain**	**Slight pain**	**Moderate pain**	**Considerable pain**	**Severe pain**
1	2	3	4	5
**2) During the past four weeks, how much trouble have you had at work or with your usual daily activities because of your leg problems?** *Circle the number that applies to you.*				
**No pain**	**Slight pain**	**Moderate pain**	**Considerable pain**	**Severe pain**

1	2	3	4	5
**3) During the past four weeks, have you slept poorly because of your leg problems, and how often?** ***Circle the number that applies to you.***				
**No pain**	**Slight pain**	**Moderate pain**	**Considerable pain**	**Severe pain**
1	2	3	4	5

**Q) During the past four weeks, how much trouble have you had carrying out the actions and activities listed below because of your leg problems?** *For each statement in the table below, indicate how much trouble you have had by circling the number that applies to you.*					
	**No trouble**	**Slight trouble**	**Moderate trouble**	**Considerable trouble**	**Could not do it**
**4) Climbing several flights of stairs**	1	2	3	4	5
**5) Crouching / Kneeling down**	1	2	3	4	5
**6) Walking at a brisk pace**	1	2	3	4	5
**7) Going out for the evening, going to a wedding, a party, a cocktail party**	1	2	3	4	5
**8) Playing a sport, exerting yourself**	1	2	3	4	5

**Q) Leg problems can also affect your spirits. How closely do the following statements correspond to how you have felt during the past four weeks?** *For each statement in the table below, circle the number that applies to you.*					
	**Not at all**	**A little**	**Moderately**	**A lot**	**Completely**
**9) I felt nervous/tense**	1	2	3	4	5
**10) I felt I was a burden**	1	2	3	4	5
**11) I felt embarrassed about showing my legs**	1	2	3	4	5
**12) I got irritated easily**	1	2	3	4	5
**13) I felt as if I was handicapped**	1	2	3	4	5
**14) I did not feel like going out**	1	2	3	4	5

Reference: Qual Life Res. 2012 Aug;21(6):1051-8.

9. Standard of care for varicose vein

The treatment of varicose veins has two primary goals: symptomatic and cosmetic. For patients with less severe varicose veins, it is recommended to avoid standing for long periods of time, crossing your legs, or doing desk work, and keeping your legs elevated above your heart when

you feel heavy or tired to help the vein flow. Prevention is best achieved by moving the entire body at least once or twice an hour, especially in occupations such as teachers, taxi drivers, and airline flight attendants, who are often in the same position for long periods of time, and by wearing compression stockings when prolonged standing is unavoidable. These methods are not curative but can help improve circulation in the veins of the legs, which can alleviate many of the symptoms that are caused by varicose veins. Compression stockings can also be used as an adjunct to other treatments and preventive measures. In addition, medication can be used to treat flavonoids (Entelon®), with the main objective of reducing symptoms.

If active treatment is required for cosmetic reasons or because of the symptoms that are caused by venous insufficiency, traditional surgical vein removal, injection sclerotherapy, endovenous closure with radiofrequency, or endovenous closure with a laser may be performed. Vein stripping involves making an incision in the skin and removing the veins that cause varicose veins as well as veins that have developed varicose veins. The procedure is very successful, with a recurrence rate of less than 10%; however, vein stripping has the disadvantage of relative scarring compared with other methods (i.e., scarring in the groin and posterior thigh). More recently, microsurgery (ambulatory varicose vein removal) has been performed, which minimizes skin incisions and uses a curved needle-like instrument to pull the veins out, leaving only small scars on the skin. Vascular laser or radiofrequency treatment is a procedure in which a laser fiber is inserted into the superficial veins (great saphenous vein, small saphenous vein, etc.) where varicose veins have developed, and the laser energy coagulates the blood and damages the blood vessel wall to close the veins. After surgery and recovery, patients can wear compression stockings and return to their daily activities the same day. This procedure is now recognized as an alternative to vein stripping, and in the United States, more than 90% of patients are treated with lasers or radiofrequency. With the introduction of ClosureFAST® (Codivien), radiofrequency treatment has overcome the existing disadvantages and can be used regardless of the size of the vessel. ClosureFAST® (Codivien) is often used in conjunction with laser treatment because the treatment effect is equivalent to laser treatment, but there is little pain after treatment. In the case of deep veins that are responsible for 90% of blood transport, surgical treatment is not possible, and only conservative treatment is possible.

10. Termination and withdrawal criteria

10.1. Criteria for stopping and dropping out of the study

1) If the patient withdraws consent
2) If during the trial period, the patient received medication or treatment without the direction of the physician in charge that may have affected the results of the study
3) If the patient fails to comply with the doctor's instructions
4) If patients who participated in the trial did not meet the inclusion and exclusion criteria
5) If adverse events that make it difficult to conduct the study occur
6) If it is deemed impossible to follow up during the trial period

7) If a patient's condition changes, and the investigator determines that it is no longer possible to participate in the trial from a safety or ethical perspective
8) If a becomes pregnant or begins nursing during the study period (from the date of written consent to the date of study discontinuation or completion of all planned study procedures)
9) Otherwise, in the judgment of the investigator, if a patient is no longer able to continue in the clinical trial

10.2. Handling of stops and dropouts

In the event of discontinuation or withdrawal from the study, the principal investigator or person in charge shall discontinue the administration of the investigational drug and record the date of discontinuation and withdrawal, reason for discontinuation and withdrawal, along with all data obtained up to the time of discontinuation and withdrawal in the supporting documents and CRF. In particular, if the patient does not visit the clinic during the study, the reason for and future progress will be followed up through e-mail, telephone, etc., and progress will be recorded in the CRF. The results of the clinical trial obtained up to the time of discontinuation or dropout may be reviewed for evaluable items during the final evaluation.

11. Data Analysis & Statistical Considerations

11.1. Sample size calculation

This is an exploratory study to determine the effectiveness of medication in improving venous reflux in patients with varicose veins, and there are limited prior studies to draw upon. Therefore, the study design is based on some assumptions, and it is not advisable to apply the same process to calculate the number of participants that are required to meet the mathematical basis for a confirmatory study when controlling for type 1 and type 2 errors.
Nevertheless, a few assumptions can be made to develop a power-calculation process similar to that of a confirmatory study. According to the literature, the duration of reflux was measured slightly differently at each point, and we calculated the number of participants based on the GSV, prox, which had the largest number of patients (Reference: J Vasc Surg. 2004 Aug;40(2):303-10.). In this study, we examined the difference between the means, but we could not find any evidence; therefore, we used the median value of the references as the mean. This assumes that n is sufficiently large (174), according to the principle of the central limit theorem, so the gap between the median and mean is not large. We also calculated the standard deviation using the range and number of n. By calculating the standard deviations of the other parameters together, we found that the standard deviation of the valve-carrying segments can be assumed to be approximately 1.8 overall. The results of the drug group are expected to improve by approximately 10% to 15%; therefore, we decided to conservatively assume an improvement of 12.5% to calculate the number of participants.

Valve-carrying segment	n	Median (s)	Range (s)	sd
GSV, prox	174	6.1	0.7-10.0	1.737
GSV, dist	84	3.5	0.7-10.0	1.921
SSV	91	4.6	0.6-10.0	1.918

In this case, assuming a normal distribution and based on a Z-test with the same variance in both groups, the following calculation result was obtained under $\alpha=0.025$, $\beta=0.2$. The calculations were performed using the PASS15. A one-tailed test was performed to determine superiority if the difference between the drug and lifestyle treatment groups was less than zero. Assuming that patients may drop out during the study, the dropout rate was set at 20% to account for dropouts in a typical clinical setting. Therefore, the total number of participants was calculated as 160, with 80 participants in each group. Considering a dropout rate of 20%, we plan to recruit 200 participants, 100 in each group, to conduct the study.

11.2. Statistical analysis plan

Categorical variables will be expressed as percentages and numbers and will be compared using the Chi-square or Fisher's exact test. Continuous variables will be presented as means, standard deviations, medians, and interquartile ranges and will be compared using an independent two-sample t-test or Wilcoxon rank sum test, while the normality of the underlying variable distribution will be estimated using histograms, skewness, kurtosis, and the Kolmogorov-Smirnov test.

The primary outcome will be presented and compared between the two groups using an independent two-sample t-test. Normality of the empirical distribution of the primary outcome will be evaluated. If this assumption is violated, a nonparametric Wilcoxon rank-sum test will be used. Secondary outcomes will also be summarized and compared between groups using independent two-sample t-tests for continuous outcomes, or chi-square test or Fisher's exact test for categorical outcomes. Considering the factors that may influence the outcome, aside from the randomly assigned intervention, we will conduct an analysis involving the adjustment of the baseline characteristics for primary and secondary outcomes. This careful analytical strategy ensures that our findings are not confounded by pre-existing differences, thereby allowing us to assess the true impact of the intervention more accurately. A planned, exploratory subgroup analysis will be performed to compare the treatment effect in patients with vericose veins with and without Entelon® medication. Unless otherwise specified, all tests will be performed at a significance level of 0.05. All analyses will be performed using SAS 9.4 (SAS Institute Inc., Cary, NC, USA). Statistical support will be provided by Nak-Hoon Son, PhD.

12. Care for the safety of the participants

12.1 Definition of safety-related terms

The safety-related terms of this clinical trial are defined in accordance with the Good Clinical Practice Act (Rules on the Safety of Drugs).

1) Definition of Adverse Event (AE)

Undesirable and unintended signs (e.g., abnormal laboratory test results), symptoms, or diseases that occur during the administration or use of a drug, etc. and are not necessarily causally related to the investigational drug.

2) Definition of Adverse Drug Reaction (ADR)

An ADR refers to a harmful and unintentional reaction caused by normal administration and use of a drug, and its causal relationship with the drug cannot be excluded. If the causal relationship with the drug is not known among the voluntarily reported adverse events, it is considered an adverse drug reaction. However, if both the reporter and the manufacturer/client determine that it is not related to the drug, it shall be excluded as an adverse drug reaction.

3) Serious AE/ADR (Serious AE/ADR)

Serious AE/ADR refers to an adverse event or adverse drug reaction that falls under any of the following categories:

① A case that results in death or is life-threatening

② A case that requires hospitalization or an extension of the hospitalization period

③ Cases that result in persistent or severe disability or diminished function

④ Cases resulting in congenital deformities or abnormalities

⑤ In addition to cases ①~④, other medically significant circumstances, such as the development of drug dependence, abuse, or blood disorders, that occur and require treatment.

The following events are not considered serious adverse reactions:

① Routine treatment or monitoring of the investigational indications that are not associated with worsening of the condition

② Procedures for protocol/disease-related investigations (however, hospitalization or prolongation of hospitalization due to complications of such procedures shall be considered an SAE)

③ Hospitalization or prolongation of hospitalization for technical, practical, or social reasons in the absence of an adverse event

④ Voluntary treatment of a pre-existing condition unrelated to the indication for which the study is being conducted

⑤ Emergency treatment or observation as an outpatient that did not result in hospitalization

⑥ Hospitalization or prolongation of hospitalization due to a plan (i.e., scheduled prior to the start of the study)

⑦ Hospitalization not for the purpose of improving or treating poor health conditions (e.g., nursing care and administrative reasons).

However, the occurrence of major ischemic and hemorrhagic events, or any hospitalization or procedure resulting from them, will not be considered a serious adverse event, given the underlying condition of the patient in this study:

- Death
- MI
- Repeat revascularization
- Stent thrombosis
- CVA/Stroke
- Bleeding (BARC 2-5)

12.2 Recording of Adverse Events

Symptoms and signs of adverse events, related actions, date of onset, severity or criticality of the adverse event, severity (maximum intensity), course, causality to the investigational product, and outcomes should be recorded in the CRF, including details of dose changes and

treatment, if necessary. Adverse events identified at previous visits should be followed up at subsequent visits, and follow-up of adverse events should be documented in the CRF.

12.3 Evaluation of Adverse Events

12.3.1 Assessing the severity of an AE

The severity of an AE is categorized according to the following criteria based on maximal intensity:

1=Mild: An AE that is barely noticeable by the patient, does not interfere with normal daily activities (functioning), and most often does not require treatment.

2=Moderate: Patients may feel discomfort and interference with normal daily activities (functioning); patients may be able to continue the study but may require treatment.

3=Severe: Extremely uncomfortable and interferes with normal daily activities (functioning), may prevent the patient from continuing to participate in the study and may require treatment or hospitalization.

12.3.2 Assessing the Causal Relationship of Adverse Events to the Investigational Product

The causal relationship to the study medicinal product will be categorized into six levels as follows and the opinion of the principal investigator or study staff will be recorded, if necessary. All but "unlikely" of the causal relationships will be evaluated as adverse drug reactions in which a causal relationship with the study drug cannot be excluded.

1) Certain

The relationship between the administration and use of the drug product is plausible and not explained by other drugs, chemicals, or concomitant conditions; the response is clinically plausible upon discontinuation of the drug product and pharmacologically or phenomenologically conclusive upon reintroduction of the drug product, as needed.

2) Fairly certain (Probable/Likely)

The temporal relationship between the administration and use of medicinal products is reasonable and does not appear to be due to other medicinal products, chemicals, or concomitant conditions. There is a clinically reasonable response to the discontinuation of the medicinal product (no re-dosing information).

3) Possible

The temporal relationship between the administration and use of the medicinal product is reasonable, but can also be explained by other medicines, chemicals, or concomitant conditions, and information on the discontinuation of the medicinal product is lacking or unclear.

4) Unlikely

A temporary case is unlikely to be causally related to the administration or use of the drug and can be reasonably explained by other drugs, chemicals, or potential diseases.

5) Evaluation difficulties (Conditional/Unclassified)

More information is needed for a proper evaluation or additional information is being reviewed.

6) Unassessable/Unclassifiable

Insufficient or conflicting information is available to make a judgment and cannot be supplemented or confirmed.

The relationship between an adverse event and the investigational drug (or other causes, progression of the underlying disease, concomitant treatment, etc.) will be determined based on how well the adverse event can be explained based on the clinical judgment of the principal investigator and staff, in terms of the following:

1) The known pharmacologic actions of the investigational drug
2) Similar actions previously observed with the investigational drug or drugs in the same class of drugs
3) Reactions that have been frequently reported to be associated with similar drugs (e.g., blood disorders)
4) Reactions that appear to be time-related (disappear when the drug is discontinued and reappear when the drug is restarted).

12.3.3 Actions Taken Regarding Adverse Events

Actions taken in response to adverse events can be categorized into those related to the investigational medicinal product and those unrelated to the investigational medicinal product as follows:

1) Investigational drug measures

① Discontinuation: Discontinuation of an investigational drug due to a specific adverse reaction

② Dose maintenance: Continued administration of an investigational drug because of an adverse event that does not require discontinuation.

③ The reason for discontinuing an investigational drug owing to an adverse event is unknown (e.g., loss of contact with the patient).

④ Not applicable: Discontinued the investigational drug for reasons other than the specific adverse event (e.g., study termination or discontinuation of the investigational drug before the adverse event occurred).

2) Other actions

① No action taken (no action taken)

② Concomitant medication taken (concomitant medication taken)

③ Non-drug therapy given (non-drug therapy given)

④ Etc (comments)

12.3.4 Outcome of the adverse event

1) Recovered/Resolved (Recovered/Resolved)
2) Recovering/Resolving(Recovering/Resolving)
3) Not recovered/Not resolved (Not recovered/Not resolved)
4) Recovered with sequelae/Resolved but with sequelae (Recovered with sequelae)
5) Fatal damage (Fatal)
6) Death may be related to AE (Death unrelated to AE)
7) Death unrelated to AE (Death unrelated to AE)
8) Unknown (Unknown)

12.3.5 Follow-up of Adverse Events

The Principal Investigator or study staff should follow patients with adverse events until the symptoms subside, abnormal clinical laboratory values return to baseline, or there is a satisfactory explanation for the observed changes. The progression of the AE should be documented in the supporting documentation and CRF.

12.3.6 Progression of Adverse Events

1) Continuous: All events except one and intermittent.
2) Intermittent: Adverse events that occur occasionally or events that occur repeatedly over a continuous period of time.
3) Single-episode: an adverse event occurred only once.

12.3.7 Reporting of Adverse Events

All AEs that occur during the study, even if unrelated to the investigational product, will be recorded in detail in the CRF, including symptoms and signs, onset/end date, severity, treatment and course, relationship with the investigational product, and significance. The collection of AEs begins after the initiation of the study medication and continues until the patient's last visit (or last contact) or early termination visit.

12.3.8 Reporting of Serious Adverse Events

If a Serious Adverse Event occurs, the Principal Investigator must notify the IRB within 72 h in accordance with the institution's SOPs.

For all serious and unexpected adverse events, the Principal Investigator must report to the IRB and the Commissioner of Food and Drug Safety in accordance with the Rules for the Safety of Drugs and Other Medicinal Products and Good Clinical Practice for Pharmaceuticals, Item 8.

Provide as complete information as possible, but at a minimum include the following information:

1) The patient to whom the SAE occurred (identification number)
2) The suspect drug
3) The source of the information (investigator)

4) Name of the SAE

Follow-up reports after the initial report will provide additional details, including an assessment of causality. Additionally, the principal investigator or study personnel will report to the IRB according to the institution's SOPs.

13. Funding Agency

Hanlim pharm.CO.LTD.

14. Study schedule

	**Baseline**	**Period**		
**Study Visit**	**V1**	**V2**	**V3**	**V4**
**Period**	**Run-in/ Screening**	**Baseline**	**Interim Assessment**	**Closing**
**Week/Day**	**-4~-2W**	**Day 0**	**6W(±14D)**	**12W(±14D)**
Written Informed Consent[a]	●			
Grant Screening No.	●			
Check inclusion/exclusion criteria	●	●		
Demographic Survey	●			
Drug administration history survey[b]	●	●		
Randomization (assignment of registration number)		●		
Lower extremity venous Doppler ultrasound examination	●		●	●
Blood test and urinalysis	●		●	●
Symptoms: VCSS, CIVIQ-14	●		●	●
Medication history survey	●	●	●	●
Adverse event investigations	●	●	●	●

a. No procedures in this study may be initiated prior to obtaining informed consent.

b. Previous medications will be investigated and recorded only for medications used within 1 month prior to obtaining informed consent.

c. Adverse events will be investigated after the first study-related procedure (i.e., date of signature of informed consent) until the last assessment date.

15. Study period

2023.07.01 ~ 2024.12.31

Patient enrollment: IRB approval date ~2024.04.31 (approximately 12 months of enrollment)

End of follow-up period:2024.07.31 (12 weeks after end of recruitment)

Analysis and report: ~2024.12.31

16. References

1. Proebstle TM, et al. The European multicenter cohort study on cyanoacrylate embolization of refluxing great saphenous veins. J Vasc Surg. 2015 Jan;3(1):2-7.

2. Gloviczki P, et al. The care of patients with varicose veins and associated chronic venous diseases: Clinical practice guidelines of the Society for Vascular Surgery and the American Venous Forum. J Vasc Surg. 2011 May;53(5 Suppl):2S-48S.

3. KVIS Mannual 2판, Chapter 10. p294.

4. Beebe-Dimmer JL, et al. The Epidemiology of Chronic Venous Insufficiency and Varicose Veins. Ann Epidemiol. 2005 Mar;15(3): 175 -184.

5. J.P. HENRIET et al. Veno-lymphatic insufficiency 4,729 Patients undergoing hormonal and procyanidol oligomer therapy. Phlebologie. 1993 Apr-Jun:46(3):313-25.

6. P. Delacroix et al. Double-blind trial of Endotelon in chronic venous insufficiency. Revue de Medecine, 7 september 1981, T22, pp1793-1802.

7. Sano A, Tokutake S, Seo A. Proanthocyanidin-rich grape seed extract reduces leg swelling in healthy women during prolonged sitting. J Sci Food Agric. 2013 Feb;93(3):457-62.

8. A Pecking, JP Desprez-Curely, G Megret, M Delaby et al. Oligomerès procyanidoliques: une alternative médicale dans le traitement du lymphoedème. ARTERES ET VEINES, 1989, Vol.8, (4):302-307.

9. Cesarone MR, Belcaro G, Rohdewald P, et al. Comparison of Pycnogenol and Daflon in treating chronic venous insufficiency: a prospective, controlled study. Clin Appl Thromb Hemost. 2006 Apr;12(2):205-12.

17. Patient Consent Form

Study name: A Prospective, Open-label, Pragmatic Randomized Controlled Trial to Demonstrate the Effect of Improving the Venous Reflux Time by Vitis Vinifera Seed Extract in Patients with Varicose Vein of Lower Extremity

Principal investigator: In Hyun Jung, M.D., PhD. Professor, Department of Cardiology,Yongin Severance Hospital, Yonsei University College of Medicine

We have asked you to participate in this clinical trial. Please take the time to read this informed consent document carefully before deciding whether to participate in this study and ensure you understand all the information in this document before deciding whether to participate. If you prefer, you may discuss your decision with a family member or doctor.

You will be in this study if you decide to participate and then voluntarily agree to do so by completing the consent form. You may decide not to participate in this study. If you decide not to participate, it will have no effect on the treatment you will receive and you will not be penalized in any way.

1. Background and purpose of clinical trials

This study is designed to determine the improvement in venous reflux time using ultrasound after taking Entelon® medication for varicose vein disease.

You have been asked to participate in the clinical study. In general, clinical research studies are conducted for medical advancement. The background of this clinical study is as follows:

Varicose veins are caused by malfunctioning valves in the veins of the legs that allow blood to return to the lower extremities. This causes blood to be collected from the lower extremities, which results in various symptoms.

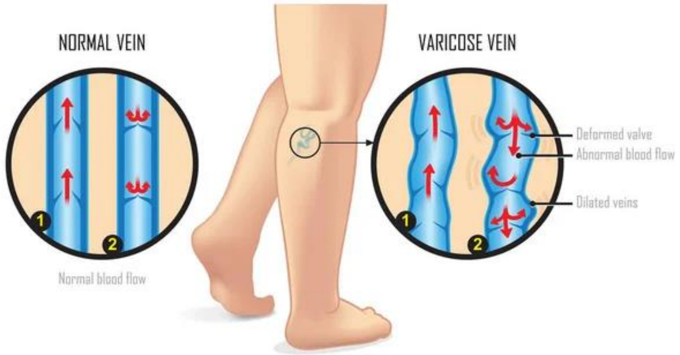

It is usually caused by venous valve dysfunction in the large trunks of superficial veins of the deep veins, such as the great saphenous vein, which flows into the deep veins from the groin area, the small saphenous vein in the pudendal region, or the penetrating vein in the calf,

whereby blood flows back up, causing the veins in the calf area to stretch. Thus, valve failure in the veins of the lower extremities is the main cause of varicose veins.

Varicose veins are usually diagnosed using Doppler ultrasound to check for reflux in the superficial (great saphenous, small saphenous, etc.) and deep (popliteal, etc.) veins, and any dilatation of the branching veins will confirm the diagnosis and determine the direction of treatment.

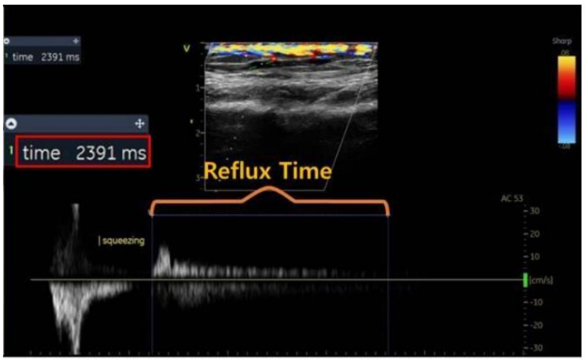

Lower limb venous Doppler ultrasound - Venous reflux time test

The treatment of varicose veins can generally be divided into conservative and invasive treatments, with conservative treatment consisting of lifestyle modifications and the use of compression stockings. Surgical treatment is indicated if conservative treatment is ineffective or interferes with activities of daily living. Symptoms can be relieved by removing the superficial veins that are refluxing due to valve failure or by inducing hardening of the causative blood vessels using radiofrequency or a laser to block reflux and prevent blood flow. However, most of these treatments are non-reimbursable and relatively expensive, resulting in a high financial burden on the patient. In addition, in the case of deep veins, surgical treatment is not possible because they are responsible for 90% of blood transport, and only conservative treatment is possible.

As a non-invasive treatment, only the intravenous activator Entelon is listed as a reimbursed drug, and its main purpose is to improve symptoms associated with venous lymphatic insufficiency (lower extremity weakness, pain, and restless legs). Although relatively inexpensive compared with surgical treatment, no objective improvement in vascular function has been demonstrated with pharmacotherapy.

The Entelon agent was a complex of grape seed extract and a natural extract. It is known for its ability to repair and improve the structure of blood vessel walls, strengthen the blood vessel walls, protect blood vessel wall components, and provide antioxidant protection by inhibiting cholesterol accumulation.

In this study, we want to determine the improvement in venous reflux using follow-up Doppler ultrasound scans in patients with varicose veins after receiving Entelon in combination with lifestyle therapy. Therefore, we are conducting a clinical trial. Your decision to participate in this study is your own and will not affect your clinical judgment regarding the treatment of this condition. Please take the time to read this information sheet and ask the investigators any

questions before deciding whether to participate.

2. **Information about investigational drugs**
   Product Name: Entelon Tablets 150mg
   Main Ingredients: Vitis vinifera seed dried extract (grape seed dried extract)
   Appearance: Yellow, round, enteric-coated tablets Storage
   Conditions: Airtight container, room temperature (1~30℃)

3. **Number of participants in the trial and duration of participation**
   This study will include 200 patients with varicose vein disease. The total expected duration of this study is approximately 2 years from the date of IRB approval. Your participation will last for 3 months.

4. **Clinical trial methods**
   The study will be 'selective' for people who have been seen by their doctors (principal investigators and staff), who have been judged to have venous reflux confirmed by Doppler ultrasound of the lower extremities, and who 'fit' the various criteria set out in this study. Pregnant and lactating women are excluded.

   If you voluntarily agree to participate in this study in accordance with the informed consent process, you will be screened for suitability for the study, and the results will determine your participation. If you are selected to participate in this study, you will undergo two lower-extremity venous Doppler ultrasound examinations. After the screening visit (Visit 1), you will not take any medications that may affect the results of the study, and you will return in 14–28 days to be randomly assigned to one of two groups: lifestyle therapy plus medication or lifestyle therapy. Randomization means that you will be assigned to either the treatment group (taking Entelon® with lifestyle therapy) or the control group (lifestyle therapy alone) with a 1:1 chance of flipping a coin and getting heads or tails.

   Patients in the treatment arm of this study will begin taking the study drug on the evening of their day 1 visit and will take it for 12 weeks (84 days). Patients in the lifestyle treatment arm will follow the following lifestyle regimen for 12 weeks:

Life Treatment
Avoid standing for long periods of time and avoid crossing your legs or doing desk legs to prevent poor circulation in your legs
When your legs feel heavy or tired, it is a good idea to keep them higher than your heart to help the veins flow.
Move your entire body at least once or twice an hour, especially if you have a job like a teacher, taxi driver, or airline flight attendant that requires you to stay in the same position for long periods of time.
If you are unavoidably on your feet or sitting for long periods of time, use compression stockings.

The lifestyle recommendations listed above are general measures for the prevention and improvement of varicose veins; however, depending on the patient's individual circumstances, it may be difficult to fulfill these recommendations. Therefore, this study's consent form recognizes that it may be difficult to fulfill lifestyle recommendations depending on the patient's individual circumstances, and the healthcare team will encourage and support the patient in fulfilling these lifestyle recommendations as much as possible during the study.

Treatment after randomization (Visit 2 through Visit 4)

Test group	Control group
Lifestyle Therapy + Entelon® 150 mg twice daily for a total of 2 tablets	Life Treatment

On visit days 6 and 12 weeks after the randomization date, you will be asked to return to the clinic to be examined by your physician.

At each visit, a test will be performed to assess efficacy and safety. There will be a total of four visits during the entire study period, as shown in the table below.

Visit 1	Visit 2	Visit 3	Visit 4
4 weeks to 2 weeks ago	0 days	6 weeks ± 14 days	12 weeks ± 14 days
Screening	Randomization	Efficacy and safety assessment (lower extremity venous Doppler ultrasound)	End-of-visit Efficacy and safety assessment (lower extremity venous Doppler ultrasound)

① Visit 1- <u>Screening Visit: Procedure and preparation for the visit to determine if you are an eligible patient for this study.</u> If you agree to participate, you will be asked to complete the screening visit tests/investigations below to assess your condition and suitability to participate.

Only identified patients are eligible to participate in the study. The screening visit is estimated to take approximately 1 hour but may take less or more time depending on the hospital's capacity. If you have test results that were obtained prior to your screening visit and the study allows for the use of these tests, they may be used as screening data for your convenience.

[Screening visit research item]

- Sign the agreement
- Review the patient's medical history and demographic information: date of birth, sex, height, weight, smoking, drinking, etc.
- Review inclusion/exclusion criteria eligibility
- Physical examination
- Investigate past or present medical history
- Investigate past or current treatments: investigate drug/non-drug (surgery, procedure, etc.) regimens to assess the presence of factors that could interfere with drug administration.

- Blood and urine tests (The tests will be conducted under the standard of care. The test results will be utilized for research purposes only, and human remains will be immediately disposed of and managed in accordance with the applicable laws.)
- Lower extremity venous Doppler ultrasound imaging: patients with venous reflux times of 0.5 seconds or more in bilateral superficial veins and 1 second or more in deep veins
- Symptom assessment: Venous Clinical Severity Score (VCSS), CIVIQ-14

② Visit 2 (Randomization Visit, Day 0)- Check inclusion/exclusion criteria

- Investigate medical/surgical history
- Physical exam and vital signs
- Randomization (assigning enrollment numbers)

③ Visit 3 (midterm assessment visit, 6 weeks from randomization visit)

- Lower extremity venous Doppler ultrasound (required)
- Symptom assessment: VCSS (Venous Clinical Severity Score), CIVIQ-14
- Check for concomitant medications (antiplatelet, antithrombotic, hyperlipidemic, etc.)
- Lifestyle-how often to wear stockings, Lifestyle+medication–check medication adherence
- Blood and urine tests (conducted under the standard of care, the results are used for research purposes only, and the human remains are immediately disposed of and managed in accordance with applicable laws)
- Monitoring adverse events

④ Visit 4 (End visit, 12 weeks from randomization visit)

- Lower extremity venous Doppler ultrasound (required)
- Symptom assessment: VCSS (Venous Clinical Severity Score), CIVIQ-14
- Check for concomitant medications (antiplatelet, antithrombotic, hyperlipidemic, etc.)
- Lifestyle-how often to wear stockings, Lifestyle+medication-check medication adherence
- Blood and urine tests (conducted under the standard of care, the results are used for research purposes only, and the human remains are immediately disposed of and managed in accordance with applicable laws)
- Monitoring adverse events

5. **Alternative treatments (other available treatments ouside of clinical trials)**

If you decide not to participate in this study, the standard of care will apply, and you can discuss this with your primary care physician. The standard of care to date is the same as that for the control group, that is, conservative treatment, including lifestyle modification and compression stockings, and a flavonoid medication (Entelon) may be used for symptomatic relief. Patients assigned to the trial arm of the study will

undergo the same tests as those in the control arm, except for the addition of the drug, Entelon.

6. Anticipated side effects, risks, and discomforts for research participants

Participants are randomly divided into the experimental and control groups. This is done to increase the reliability of the results. With randomization, participants cannot predict the group to which they will be assigned and all tests will be performed by qualified professionals to minimize discomfort. The tests performed in this study (such as ultrasound and blood draws) may cause some discomfort, and some participants may have less favorable outcomes than others. Therefore, if you are interested in participating in this study, you must fully understand the risks and discomfort associated with randomization and the risks and discomfort that may occur with the tests that are performed in this study. The Entelon medication is known to cause rare skin reactions such as urticaria, skin rash with or without itching, photosensitivity, eczema, and gastrointestinal adverse reactions such as stomach pain, nausea, and diarrhea, which resolve upon discontinuation. These complications have not been reported in large numbers, nor have they been reported as too dangerous for use of the drug in the general patient population owing to its lack of safety. Therefore, this study aimed to verify the clinical effectiveness of Entelon, a drug that is widely and safely used for varicose vein disease.

7. Who pays for testing and guidance for care

The cost of the two lower extremity venous Doppler ultrasound examinations that are required for this study will be addressed by the investigator. However, patients will undergo blood and urine tests at the first visit, which are not related to this study, according to the standard of care guidelines. Additionally, patients are responsible for the treatment of common varicose veins and other tests, such as blood tests, under the insurance regulations of the National Health Insurance Service. Therefore, patients are responsible for the cost of the drug, Entelon, under the insurance regulations of the National Health Insurance Service.

8. Honorariums for clinical trial participation

The researcher will pay the cost of the Doppler ultrasound examination of the lower extremity veins necessary to conduct this study. In addition, as an example of the loss of time and inconvenience that may be caused by participating in the study, the researcher will provide transportation reimbursement per visit from the third visit, depending on the duration of participation (50,000 won per visit for a total of 100,000 won when two visits are completed). If you drop out of the study or withdraw your consent during the study, transportation reimbursement for the last visit will be partially paid. However, you will be responsible for hospitalization and medical expenses due to illnesses or accidents that occurred as unrelated to the conduct of this study, because they are covered under standard medical care.

9. Compensation and remedies in the event of harm

The cost of treatment for common varicose veins and all tests, such as blood tests, will be borne by the patients under the insurance regulations of the National Health Insurance Service. Therefore, the cost of Entelon is borne by the patients under the insurance regulations of the National Health Insurance Service. In addition, the costs of testing, treatment, and compensation for adverse events related to the drugs used

in this study will be based on the victim compensation protocol. In the event of damage that is caused by participating in the study, we will proceed with medical treatment and compensation based on insurance and victim compensation protocols. Upon your request, we will provide you with a copy of the Victim Compensation Protocol. In the event of damage, and if you have any questions regarding this study, please contact Prof. In-Hyun Jung at (031)5189-8756.

10. What the recipient must do

While you are participating in the clinical trial, you must comply with the following:

- You must maintain the scheduled visits and tests for this study. If you are unable to maintain your appointment, contact your doctor or staff.
- You should tell your study doctor or staff about all side effects and abnormalities that occur during the study, even if you do not think they were caused by your participation in the study.
- You must tell your study doctor or staff about any other doctor visits or hospitalizations. You cannot take medications that affect the effectiveness of the study medication together, so you should not use any medications other than this study medication (by mouth, by injection, or by skin application). Be sure to contact your study doctor or staff in advance.
- You may not participate in any other clinical trials while participating in this study.

11. Benefits of participating in a clinical study

Although there is no guarantee that participation in this study will directly benefit you, it may help other patients by determining whether the investigational drug used in this study improves varicose vein disease.

12. Freely giving the withdrawing consent to participate in research

Your participation in this study is voluntary, and if you do not agree to participate, you will not be penalized in any way in your future care. You are free to stop participating in this study at any time, and you will not be penalized in any way in your future care.

13. Stop, drop out

Your study doctor may decide to exclude you from this study

- If you request to withdraw from the study during the study period
- If an investigational drug appears to be medically harmful to you
- If you fail to comply with procedures required by the clinical trial
- If you are found not to meet the requirements of the study
- If the trial is canceled

If this happens, we will talk to you about the reasons, and you can ask you to stop participating at any time, even if you have already begun receiving the investigational drug. If you are removed from the study, you will continue receiving treatment according to the standard of care.

14.Information Collection, Use, and Disclosure

By signing this consent form, you will provide consent to the collection and use of your personal (sensitive) information.

1) Purpose of collecting and using personal information

This study will use your personal (sensitive) information to learn about the effectiveness and safety of lifestyle treatment versus lifestyle + medication in varicose veins. You have the right to refuse consent for the collection or use of your personal information; you will not be penalized for doing so, but you will not be able to participate in this study.

2) What personal (sensitive) information that we want to collect

**Your personal and sensitive information that we collect**	
**Personal information**	**Full name, sex, age, hospital number, phone number, e-mail**
**Sensitive information**	**Health-related, such as medical records and materials generated in the course of clinical research.**

3) Period of retention and use of personal information

Your personal (sensitive) information will be properly managed in accordance with the Personal Information Protection Act and will be destroyed in accordance with Article 16 of the Enforcement Decree of the Personal Information Protection Act three years after the end of the study.

4) Your personal information will not be provided to third parties and will only be used for this study.

5) You are free to decide whether to accept the collection, use, or provision of personal (sensitive) information. If you do not accept the collection, use, and provision of your personal (sensitive) information, there will be no disadvantages to your treatment or prescription.

15.Confidentiality of records

Any records that may identify you will be kept confidential, and your identity will remain confidential if the results of the study are reported, published, or presented. By signing this consent, you or your representative authorizes the study monitor, the person conducting the inspection, the review committee, and the Director of the Ministry of Food and Drug Safety to access your medical records in accordance with applicable laws and regulations to verify the quality of the study procedures and

materials, provided that your identity is kept confidential; if the results of the study are published, your identity will remain confidential.

16.Voluntariness of joining/withdrawing

As explained earlier, you may or may not participate in the study, and you may stop participating at any time afterward. If you wish to leave the study, please inform your doctor verbally or in writing. In addition, if you withdraw your consent, your participation in the study will end and the research team will not collect any further information from you about the study. If you decide not to participate in the study or drop out, you will not be penalized in any way, and there will be no loss of any benefits to which you may have been entitled.

17.Contact

If you have any questions about this study or if you have a study-related injury, please contact the doctor listed below.

Investigator Full Name: Inhyun Jung, Department of Cardiology, Yongin Severance Hospital

Researcher Address : 363 Dongbaekjukjeon-daero, Giheung-gu, Yongin-si, Gyeonggi-do, 16995, Korea

☎ : 031-5189-8756

If you have any questions about your rights as a participant, you can talk to the researcher or contact us using the following numbers:

Yongin Severance Hospital Research Review Committee ☎ 031-8189-8891~2
Yongin Severance Hospital Clinical Research Protection Center ☎ 031-5189-8893

Patient Consent Form

Study Title: A Prospective, Open-label, Pragmatic Randomized Controlled Trial to Demonstrate the Effect of Improving the Venous Reflux Time by Vitis Vinifera Seed Extract in Patients with Varicose Vein of Lower Extremity

※ I have had time to think about this and understand the following and agree to participate voluntarily.

- I have read this informed consent document and have been fully informed and understand the purpose, methods, expected benefits, possible risks, availability and content of other treatments, and management of my health information.
- I understand the description of the collection, use, and provision of personal (sensitive) information for research purposes.

- I □ agree □ disagree to the collection and use of my personal information.
- I □ agree □ disagree to the collection and use of my sensitive information.

- I asked all the questions I had, and I felt like I had enough answers.
- I understand that if I agree to participate in this study, I can withdraw at any time, and that I can receive other appropriate treatment after withdrawal.
- I received one copy of the explanatory statement and completed consent form.

Patient Name		Signature		Signature date	

Also known as a 'guardian' or 'legally acceptable representative (LAR)'

* LAR Name (if needed)		Signature		Signature date	

(Relationships with patient:)

(Reasons for proxy consent:)

Observer Name (if needed)		Signature		Signature date	

Full name of the reasercher Name		Signature		Signature date	

VICTORY TRIAL
